# Circulating levels of blood biomarkers and risk of benign prostatic hyperplasia: Results from two large cohorts in Europe and East Asia

**DOI:** 10.7189/jogh.14.04242

**Published:** 2024-11-22

**Authors:** Shengzhuo Liu, Xiaoyang Liu, Pan Song, Luchen Yang, Zhenghuan Liu, Jing Zhou, Linchun Wang, Xin Yan, Kai Ma, Yunfei Yu, Xianding Wang, Qiang Dong

**Affiliations:** 1 Department of Urology, Institute of Urology, West China Hospital, Sichuan University, Chengdu, China; 2 Kidney Transplantation Center, West China Hospital, Sichuan University, Chengdu, China

## Abstract

**Background:**

As one of the most prevalent chronic non-communicable diseases affecting aging males, the burden of benign prostatic hyperplasia is growing over the world. Our study aims at investigating the potential relationships between various blood biomarkers and benign prostatic hyperplasia (BPH) in middle-aged and older men in European and East Asian population cohorts.

**Methods:**

We included 229 022 male adults from the UK Biobank (UKB) and 20 284 male adults from the China Health and Retirement Longitudinal Study (CHARLS) in this study. Forty-four blood biomarkers in UKB cohort and 16 blood biomarkers in the CHARLS cohort were analysed to examine their association with benign prostatic hyperplasia. Cox, logistic analyses and restricted cubic spline models were used to investigate linear and nonlinear longitudinal associations.

**Results:**

In our research, elevated high-density lipoprotein cholesterol showed significant associations with a decreased risk of benign prostatic hyperplasia, and these associations remained significant after accounting for potential covariates both in UKB cohort (hazard ratio (HR) = 0.83; 95% CI = 0.79–0.88, *P* < 0.001) and CHARLS cohort (odds ratio (OR) = 0.992; 95% CI = 0.985–0.999, *P* = 0.033). Apolipoprotein A was also found to be inversely associated with BPH (HR = 0.76; 95% CI = 0.70–0.81, *P* < 0.001). L-shaped relationships were discovered between level of high-density lipoprotein cholesterol and apolipoprotein A with incidence of benign prostatic hyperplasia.

**Conclusions:**

This large prospective biomarker-based study highlights that high-density lipoprotein (HDL) cholesterol and apolipoprotein A are significant protective factors against the development of BPH, with L-shaped associations suggesting an optimal protective range. In contrast, biomarkers related to glucose metabolism, inflammation, and hormone levels were not found to significantly influence BPH progression. Our findings support the potential involvement of lipid biomarkers in the early stages of BPH development, suggesting that future strategies should prioritise lipid-related pathways in the prevention and management of BPH.

Benign prostatic hyperplasia (BPH) is distinguished by symptoms such as urinary incontinence and retention, standing as the most prevalent urologic condition among older men. Its onset typically becomes evident upon reaching the age of 45, with a discernible increase in incidence over time, with approximately 25% of men in their fifties, 33% among those in their sixties, 50% of men in their eighties [1,2]. Bladder outlet obstruction (BOO) manifests when the prostate gland exerts physical pressure on the urethra due to an expansion of stromal and epithelial cells. This condition is often associated with lower urinary tract symptoms, which encompass a spectrum of obstructive and irritative voiding symptoms [3].

Despite its widespread prevalence, the aetiology of BPH is not well understood. Previous studies have focused predominantly on sex steroid hormones. Nevertheless, it is worth noting that in addition to androgens and genetic predisposition, there is an increasing number of studies suggesting that circulating levels of blood biomarkers may also affect the risk of BPH and perhaps influence progression [4]. Prior studies have demonstrated an association between dyslipidaemia, hyperglycaemia, and metabolic syndrome with an elevated risk of BPH [5–7]. The study of J Hammarsten indicated that fasting insulin, systolic blood pressure, and high-density lipoprotein (HDL) are closely related to benign prostatic hyperplasia [8]. Prior studies have indicated a significant correlation between abnormal blood biomarkers in the male circulatory system and an elevated risk of BPH morbidity [9].

The widespread prevalence of benign prostatic hyperplasia among middle-aged and older males not only exacerbates their distress but also imposes substantial economic and medical burdens on society, thus impacting their overall quality of life [10]. To this end, the present study aims to examine the correlation between circulating blood biomarker levels and the likelihood of developing BPH in male participants enrolled in both the UK Biobank and CHARLS study. Our discoveries could potentially offer insights into the creation of innovative strategies designed to mitigate the onset of BPH and improve the well-being of the older population.

## METHODS

### Data resource and study population

Our study draws upon data from two extensive cohort studies: the UK Biobank (UKB) and the China Health and Retirement Longitudinal Study (CHARLS) databases. The UK Biobank study is a population-based prospective investigation designed to collect data on the determinants of diseases prevalent during middle and older adulthood. Initiated between 2006 and 2010, approximately 500 000 men and women aged 40 to 69 years, were recruited from various regions across England, Wales, and Scotland. Participation in the study was drawn from individuals registered with the UK's National Health Service (NHS). Further details on the protocols employed in the UK Biobank study can be accessed at https://www.ukbiobank.ac.uk/media/gnkeyh2q/study-rationale.pdf. The CHARLS study, accessible at https://g2aging.org/, offers longitudinal data that comprehensively covers diverse factors including socio-economic status and health, derived from a national sample of elderly individuals in China. CHARLS utilised a multistage stratified probability sampling methodology to obtain a representative sample of the population. The study protocol of CHARLS received ethical approval from the Ethics Committee of Peking University Health Science Center. For an in-depth exposition of the survey methodology, please refer to previous publications on the CHARLS survey design [11].

**Figure 1:**
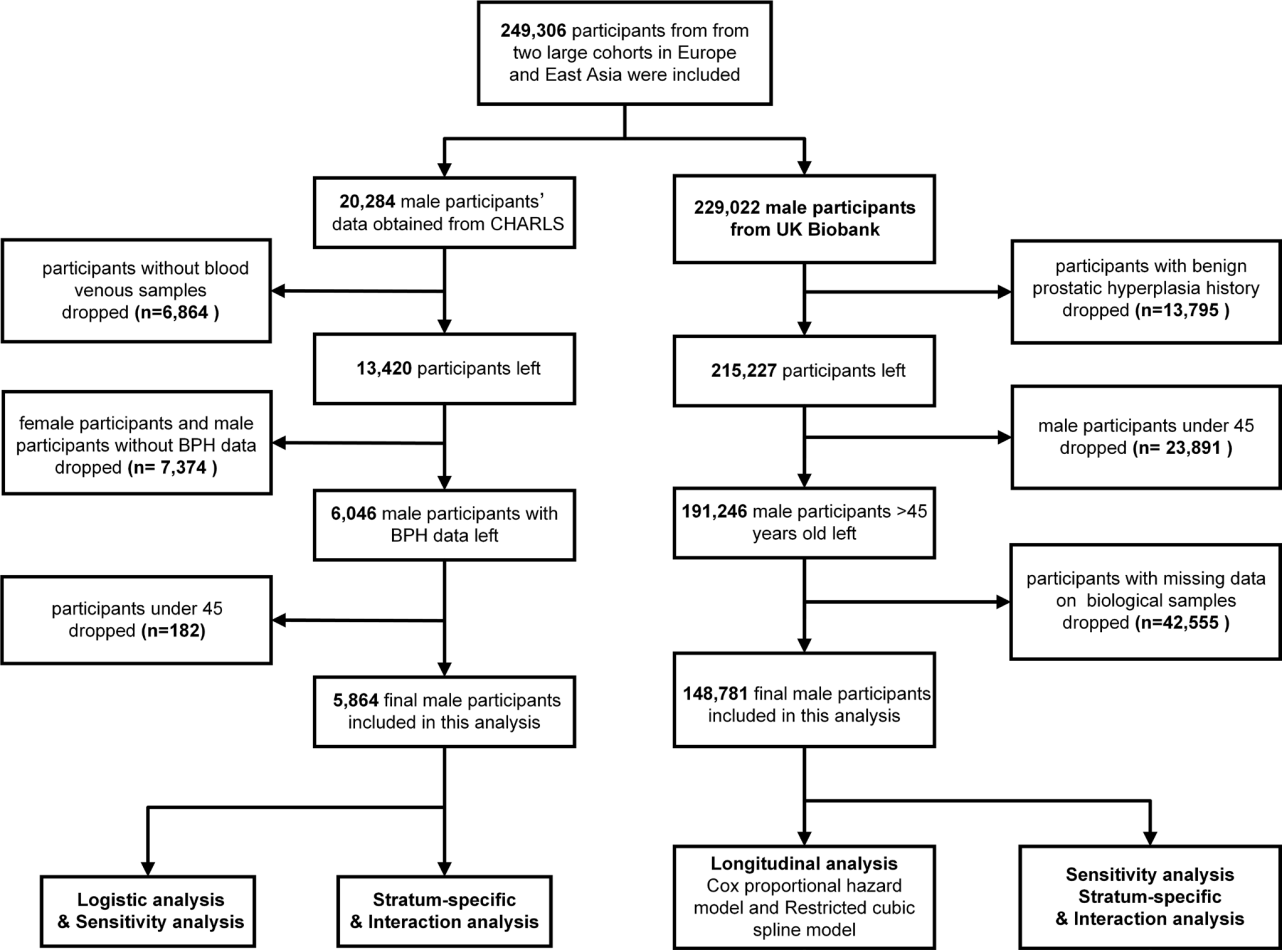
Flowchart of the total participants selection and research design in the UKB and CHARLS cohort. BPH – benign prostatic hyperplasia, CHARLS – China Health and Retirement Longitudinal Study, UKB – UK Biobank.

In the current study, a total of 229 022 men were enrolled in the UK Biobank study, and a total of 13 795 participants who had a history of BPH at the time of recruitment and 23 891 participants under 45. Participants with a documented history of BPH at the time of recruitment (n = 13 795) and those aged under 45 years (n = 23 891) were excluded from the analysis. Moreover, after excluding 42 555 participants with missing data on biological samples, In the CHARLS cohort, a total of 20 284 participants were surveyed during CHARLS Wave 3, with 13 420 consenting to provide venous blood samples. In our study, female individuals and participants without BPH information or blood markers were excluded (n = 7374). Additionally, participants under 45 were also excluded (n = 182). Ultimately, a total of 5864 adult males were finally enrolled. Additional information regarding the criteria for participant inclusion and exclusion is detailed in **Figure 1**.

### Definition of biomarkers and benign prostatic hyperplasia diagnosis

In the UKB cohort, non-fasting blood samples were obtained collected the baseline [12]. Comprehensive information regarding the handling and storage procedures of these specimens has been previously published at https://biobank.ndph.ox.ac.uk/showcase/showcase/docs/serum_biochemistry. Benign prostatic hyperplasia was diagnosed by the International Classification of Diseases 10th revision (ICD-10) codes N40. The first occurrences of BPH outcomes were generated by mapping primary care data, hospital admissions data, death register records, self-reported medical conditions and other sources (Field 132072). Follow-up visits lasted from the attendance at the assessment centre (Field 53) to the first diagnosis of BPH, death (Field 40000), or the last available date from the hospital inpatient data or primary care data (https://biobank.ndph.ox.ac.uk/showcase/exinfo.cgi?src=Data_providers_and_dates).

In CHARLS cohort, a total of 13 420 blood samples with a response rate of 64% were collected for analysis. Blood samples underwent a two-phase analytical process. At the outset, local health centres performed a thorough analysis of CBC, haemoglobin, WBC, platelet, and mean corpuscular volume. Following this, the collected samples were transported to the central laboratory, where additional analyses were conducted. These analyses included measurements of high-sensitive C-reactive protein (hs-CRP), haemoglobin A1c (HbA1c), total cholesterol, HDL cholesterol, low-density lipoprotein (LDL) cholesterol, triglycerides, glucose, blood urea nitrogen, creatinine, uric acid, and cystatin C [13]. Benign prostatic hyperplasia symptoms assessed by asking the following question: ‘Have you ever been diagnosed with a prostate illness, such as prostate hyperplasia (excluding prostatic cancer)?’ We defined the respondent as having BPH problems if he answered positively to the query.

### Covariates assessment

In the UK Biobank cohort, demographic, medical, and lifestyle information was gathered through self-reported questionnaires and measurements. These questionnaires covered various factors, including physical activity, sleep patterns, medication usage, alcohol consumption, and smoking history. Covariates selected as potential confounders were determined based on their documented associations as reported in prior literature [14–16]. Relevant covariates were measured at baseline. Demographic variables included age (quantified in units of years and divided into three groups according to the boundary of 50 and 60) and ethnic background (White, Asian or Asian British, Black or Black British, mixed and other ethnic group). Socioeconomic variables included socioeconomic status (quintiles of Townsend index of deprivation) and education (A levels/AS levels or equivalent, College or University degree, Certificate of Secondary Education (CSEs) or equivalent, National Vocational Qualifications (NCQ)/Higher National Diploma (HND)/Higher National Certificate (HNC) or equivalent, O levels/General Certificate of Secondary Education (GCSEs) or equivalent, Other professional qualifications, none of the above). Lifestyle variables included alcohol intake frequency (never, <3 times/week, ≥ 3 times/week), smoking history (no/yes), and body mass index (underweight, normal, overweight, and obesity according to the cut-off value of 18.5 kg/m^2^, 25 kg/m^2^ and 30 kg/m^2^). duration of walks (0, 0–15, 15–30, 30–60, 60–180, ≥180-minute/d), sleep duration (<7, 7–10, ≥10 hours/d); blood pressure (normal, pre-hypertension, grade 1, grade 2, grade 3) and medication intake (blood pressure medication; cholesterol lowering medication; Insulin; mixed; none of the above). Simultaneously, we adjusted for covariates in the CHARLS cohort similar to those in the UKB cohort (although there are some differences due to the database design). Participants were stratified into distinct groups based on various covariates: education (less than lower secondary, upper secondary/vocational training, and tertiary education); marital status (married or partnered, and separated, divorced, widowed, or never married); residential locations (rural or urban); smoking history (yes/no); drinking history(yes/no). Moreover, several disorders were identified as potential confounders based on the questionnaire ‘Have you been diagnosed with listed conditions by a doctor?’ The study encompassed preceding medical conditions including lung diseases, stroke, hypertension, psychological disorders, arthritis, liver or kidney disease, asthma and gastrointestinal disorder. Multivariate Imputation by Chained Equation (MICE) were employed to impute missing covariate variables in both large cohort study, specifically utilising Random forest imputations method [17].

### Statistical analysis

Standard deviations and means serve as measures of central tendency and dispersion for continuous variables that follow a normal distribution. Conversely, the interquartile range and median are employed to represent continuous variables exhibiting a skewed distribution. Rates and percentages are utilised to depict categorical variables. The Kruskal-Wallis test was employed to determine *P*-values for continuous variables exhibiting a skewed distribution, while χ^2^ tests were used to analyse categorical data.

As for UK biobank cohort, we estimated hazard ratios (HRs) and 95% confidence intervals (CIs) to assess the association of biomarkers with the risk of BPH. Cox proportional hazards regression models were utilised, with follow-up time employed as the time scale, which was defined from the baseline enrolment visit date until the occurrence of BPH diagnosis, date of death, date of loss to follow-up, or the end of the cohort's follow-up period, whichever event transpired first (https://biobank.ndph.ox.ac.uk/showcase/exinfo.cgi?src=Data_providers_and_dates). Biomarkers were analysed as continuous variables, and the proportional hazards assumptions were evaluated using tests based on Schoenfeld residuals. The preliminary model analysis investigated the relationship between all the potential blood markers and risk of BPH. Then we excluded biomarkers with relatively weak correlations (albeit statistically significant due to the large sample size) and the biomarkers that did not satisfy the proportional hazards assumption. We then performed multivariable Cox Regression models for the screened blood biomarkers. Model I was adjusted for demographic variables (age groups and ethnic background) and socioeconomic variables (Townsend index of deprivation and education). Model II added lifestyle variables including alcohol intake frequency, smoking history, and body mass index, duration of walks, sleep duration, blood pressure and medication intake. We utilised restricted cubic spline models in conjunction with Cox proportional hazards models to examine potential nonlinear relationships, while adjusting for covariates as outlined in Model II. Potential nonlinearity was initially detected using scatter plots and subsequently assessed with a likelihood ratio test, comparing a linear model to a model that included both linear and cubic spline term. The biomarkers were then analysed categorically, with the lowest quartile (Q1) of each biomarker serving as the reference category for sensitivity analysis. We also performed subgroup analyses to estimate potential modification effects according to all potential covariates.

In the CHARLS cohort, we conducted a multivariate logistic regression analysis to explore the relationship between blood biomarkers and BPH at a cross-sectional level. This analysis encompassed adjustments for covariates to mitigate the potential impact of additional confounding variables on the study outcome. Age, socioeconomic variables and underlying diseases were sequentially included in the sharing model for adjustment. Interaction analyses were conducted to assess the diversity in the correlation between blood biomarkers and BPH. Subgroup analyses were carried out using stratified logistic regression models and the *P*-value for interaction was determined via the log-likelihood ratio test

In this study, a significance level of 0.05 was utilised to determine statistical significance for all findings. All statistical analyses were conducted using *R* version 4.2.2

## RESULTS

### Baseline characteristics

In the UKB cohort, baseline characteristics stratified by BPH status are demonstrated in **Table 1**. The primary analyses included 148 781 participants without BPH from the UK Biobank. Over a median follow-up period of 13.80 years (interquartile range = 12.94–14.55), a total of 17 633 men were finally diagnosed with BPH. Men who developed BPH exhibited a higher propensity for lower socioeconomic status or educational attainment, tobacco abstinence or smoking habits, elevated body mass index (BMI), reduced physical activity levels, higher blood pressure, and a history of previous medication usage. Circulating levels of blood biomarkers stratified by the presence or absence of BPH are detailed in Table S1 in the **Online Supplementary Document**.

**Table 1:** Baselines characteristics of the participants enrolled*

Category/unit		BPH		*P*-value
		**Yes**	**No**	
		**n = 17 633**	**n = 131 148**	
**Follow-up duration, MD (IQR), year**		7.79 (4.07–10.84)	13.96 (13.25–14.64)	<0.001
**Age, MD (IQR), year**		62.00 (58.00–66.00)	58.00 (52.00–63.00)	<0.001
**Age class (%)**				
<50 y		915 (5.24)	21 683 (16.61)	<0.001
<60 y		5053 (28.94)	50 057 (38.35)	
≥60 y		11 490 (65.82)	58 792 (45.04)	
**Townsend† (%)**				
≤−3.96		3672 (20.85)	26 721 (20.40)	<0.001
≤−2.81		3729 (21.17)	26 430 (20.18)	
≤−1.34		3636 (20.65)	26 394 (20.15)	
≤1.35		3337 (18.95)	26 236 (20.03)	
≥1.35		3238 (18.39)	25 217 (19.25)	
**Qualifications (%)**				
A levels/AS levels or equivalent		1606 (9.11)	13 815 (10.53)	<0.001
College or University degree		5609 (31.81)	44 202 (33.70)	
CSEs or equivalent		567 (3.22)	6912 (5.27)	
None of the above		3961 (22.46)	23 275 (17.75)	
NVQ or HND or HNC or equivalent		1788 (10.14)	12 419 (9.47)	
O levels/GCSEs or equivalent		3182 (18.05)	24 390 (18.60)	
Other professional qualifications e.g: nursing, teaching		920 (5.22)	6135 (4.68)	
**Ethnic background (%)**				
Asian or Asian British		364 (2.06)	2781 (2.12)	0.406
Black or Black British		168 (0.95)	1367 (1.04)	
Mixed		61 (0.35)	521 (0.40)	
Other ethnic group		129 (0.73)	855 (0.65)	
White		16 911 (95.91)	125 624 (95.79)	
**Smoking status (%)**				
No		7903 (45.03)	63 365 (48.48)	<0.001
Yes		9647 (54.97)	67 346 (51.52)	
**Alcohol intake frequency (%)**				
Never		1269 (7.20)	7456 (5.69)	<0.001
<3 times/week		7482 (42.46)	53 183 (40.58)	
≥3 times/week		8871 (50.34)	70 415 (53.73)	
**BMI (%)**				
Underweight		33 (0.19)	318 (0.24)	<0.001
Normal		4033 (23.00)	32 126 (24.60)	
Overweight		8767 (50.00)	64 735 (49.56)	
Obesity		4702 (26.81)	33 436 (25.60)	
**Duration of walks (%)**				
<15 min/d		1411 (9.17)	9712 (8.40)	<0.001
<30 min/d		3777 (24.55)	28 550 (24.70)	
<60 min/d		4567 (29.68)	33 780 (29.23)	
<180 min/d		4392 (28.54)	33 054 (28.60)	
≥180 min/d		1240 (8.06)	10 483 (9.07)	
**Sleep duration (%)**				
<7 h		4312 (24.57)	32 373 (24.78)	<0.001
<10 h		12 852 (73.24)	96 020 (73.50)	
≥10 h		383 (2.18)	2240 (1.71)	
**Blood pressure (%)**				
Normal		1785 (10.13)	13 948 (10.64)	<0.001
High-normal		1795 (10.19)	13 845 (10.56)	
Grade 1		4291 (24.35)	34 386 (26.23)	
Grade 2		9209 (52.27)	64 959 (49.56)	
Grade 3		539 (3.06)	3939 (3.01)	
**Medication history (%)**				
Blood pressure medication		2276 (12.91)	13 262 (10.11)	<0.001
Cholesterol lowering medication		1895 (10.75)	10 779 (8.22)	
Insulin		33 (0.19)	233 (0.18)	
Mixed		3655 (20.73)	20 367 (15.53)	
None of the above		9774 (55.43)	86 507 (65.96)	

BMI – body mass index, BPH – benign prostatic hyperplasia, CSEs – Certificate of Secondary Education, GCSE – General Certificate of Secondary Education, HNC – Higher National Certificate, HND – Higher National Diploma, IQR – interquartile range, NVQ – National Vocational Qualifications, x̄ – mean

*Median and IQR are used to describe continuous data with a skewed distribution. Rates and percentages are used to represent categorical variables. The K-W test was used to calculate *P*-values for continuous variables with skewed distributions, while χ^2^ tests were used to assess categorical data.

^†^Social economic status represented by the Townsend deprivation index.

### Circulating levels of biomarkers and risk of BPH

**Figure 2:**
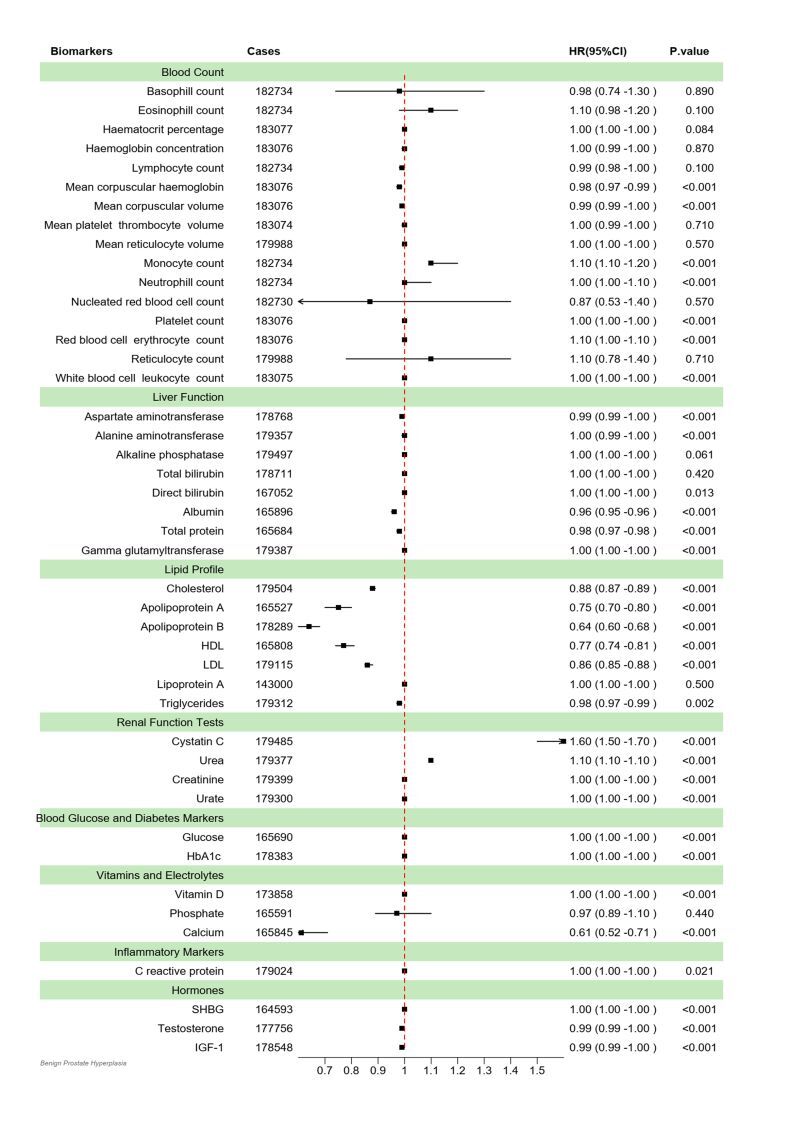
Hazard ratios (HR) and 95% confidence intervals (CI) for the association between 44 blood biomarker levels and the risk of benign prostatic hyperplasia (BPH) in UK Biobank cohort (UKB).

**Figure 3:**
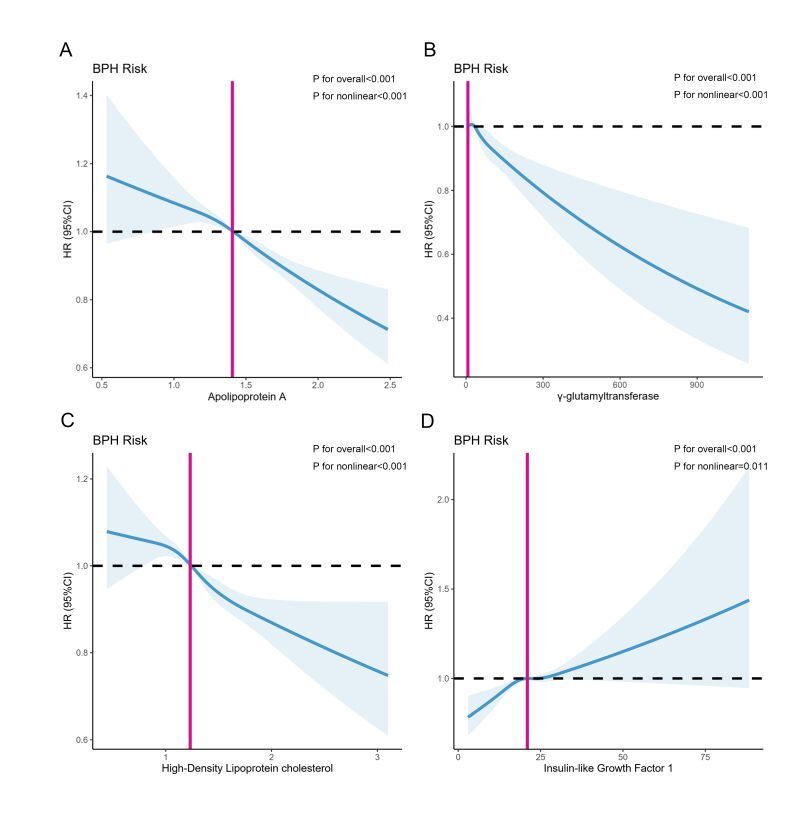
Nonlinear associations between circulating levels of biomarkers and risk of benign prostatic hyperplasia (BPH) in the UK Biobank (UKB) cohort. Restricted cubic spline models fitted for Cox proportional hazards models for four significant nonlinear associations. **Panel A.** Apolipoprotein A (g/L). **Panel B**. Gamma glutamyl transferase(U/L). **Panel C.** High-density lipoprotein (mmol/L). **Panel D.** Insulin-like growth factor 1 (nmol/L)). Results were adjusted for demographic variables, socioeconomic variables and lifestyle variables.

In the UKB cohort, the correlation between 44 blood biomarkers and the risk of prostatic hyperplasia was shown in **Figure 2**. We observe that the associations between blood cell count; blood glucose and diabetes markers; inflammatory markers and BPH demonstrated relatively modest correlations, albeit statistically significant owing to the large sample size. In the context of liver function biomarkers, albumin (HR = 0.96; 95% CI = 0.95–0.96, *P* < 0.001) and total protein (HR = 0.98; 95% CI = 0.97–0.98, *P* < 0.001) exhibited a modest inverse association with BPH. Among the biomarkers of lipid profile, cholesterol (HR = 0.88; 95% CI = 0.87–0.89, *P* < 0.001), apolipoprotein A (HR = 0.75; 95% CI = 0.70–0.80, *P* < 0.001), apolipoprotein B (HR = 0.64; 95% CI = 0.60–0.68, *P* < 0.001), HDL (HR = 0.77, 95% CI = 0.74–0.81, *P* < 0.001), LDL (HR = 0.86; 95% CI = 0.85–0.88, *P* < 0.001), and triglycerides (HR = 0.98; 95% CI = 0.97–0.99, *P* < 0.001) exhibited negative associations with the incidence of BPH. Additionally, the elevated level of cystatin C (HR = 1.60; 95% CI = 1.50–1.70, *P* < 0.001) and urea (HR = 1.10; 95% CI = 1.10–1.10, *P* < 0.001) demonstrate a significant positive association with benign prostatic hyperplasia. Concurrently, a negative correlation was observed between calcium level and BPH (HR = 0.61; 95% CI = 0.52–0.71, *P* < 0.001). At the same time, we included indicators that have been reported to have an impact on prostatic hyperplasia in the previous literature, including gamma glutamyltransferase, triglycerides, glucose, HbA1c, hormones (sex hormone binding globulin (SHBG), testosterone, and insulin-like growth factor 1 (IGF-1)). Then multivariable Cox Regression models were performed on aforementioned blood biomarkers and those unsatisfied with Proportional hazards assumptions were excluded from the main analysis. Finally, a total of nine blood biomarkers were finally included in main analysis (**Table 2**), and after controlling for demographic and socioeconomic variables, no significant associations were observed between glucose, testosterone, triglycerides, and the risk of BPH. After controlling for all potential covariates including demographic variables, socioeconomic variables and lifestyle variables, only apolipoprotein A (HR = 0.76; 95% CI = 0.70–0.81, *P* < 0.001), HDL (HR = 0.83; 95% CI = 0.79–0.88, *P* < 0.001), gamma glutamyl transferase (HR = 0.99; 95% CI = 0.99–1.00, *P* < 0.001), and IGF-1 (HR = 1.00; 95% CI = 1.00–1.01, *P* < 0.001) remained highly correlated with incidence of BPH. Restricted cubic spline models fitted for Cox proportional hazards models for above 4 significant nonlinear associations was shown in **Figure 3**. L-shaped relationships were discovered between apolipoprotein A (*P* for overall <0.001; *P* for nonlinear <0.001), gamma glutamyl transferase (*P* for overall <0.001; *P* for nonlinear <0.001), and HDL (*P* for overall <0.001; *P* for nonlinear <0.001) with incidence of BPH. Insulin-like growth factor 1 (*P* for overall <0.001; *P* for nonlinear = 0.011) had a S-shaped relationship with incidence of BPH.

**Table 2:** Hazard ratios and 95% confidence intervals for the association between blood biomarker levels and the risk of Benign prostatic hyperplasia in the UKB cohort

Biomarkers	HR	95% CI	*P*-value
Model I*			
Apolipoprotein A	0.74	0.70–0.79	<0.001
Gamma glutamyl transferase	1.00	1.00–1.00	<0.001
Glucose	1.00	1.00–1.00	<0.001
HbA1c	1.00	1.00–1.00	<0.001
HDL cholesterol	0.77	0.73–0.81	<0.001
IGF-1	0.99	0.99–1.00	<0.001
SHBG	1.00	1.00–1.00	<0.001
Testosterone	0.99	0.99–1.00	<0.001
Triglycerides	0.98	0.97–0.99	0.002
**Model II†**			
Apolipoprotein A	0.66	0.62–0.71	<0.001
Gamma glutamyl transferase	1.00	1.00–1.00	<0.001
Glucose	1.00	0.97–1.01	0.555
HbA1c	1.00	1.00–1.00	0.009
HDL cholesterol	0.75	0.71–0.78	<0.001
IGF-1	1.01	1.00–1.01	<0.001
SHBG	1.00	1.00–1.00	<0.001
Testosterone	1.00	1.00–1.00	0.597
Triglycerides	1.00	0.98–1.01	0.573
**Model III‡**			
Apolipoprotein A	0.76	0.70–0.81	<0.001
Gamma glutamyl transferase	0.99	0.99–1.00	<0.001
Glucose	0.98	0.97–1.00	0.003
HbA1c	1.00	1.00–1.00	0.125
HDL cholesterol	0.83	0.79–0.88	<0.001
IGF-1	1.00	1.00–1.01	<0.001
SHBG	0.99	0.97–1.00	0.066
Testosterone	1.00	1.00–1.01	0.086
Triglycerides	0.99	0.97–1.00	0.053

CI – confidence interval, HbA1c – haemoglobin A1c, HDL – high-density lipoprotein, HR – hazard ratio, IGF-1 – insulin-like growth factor 1, SHBG – sex hormone binding globulin, UK – UK Biobank

*Model I adjusted for none.

†Model II adjusted for demographic variables (age class; ethnic background) and socioeconomic variables (socioeconomic status; education).

‡Model III adjusted for demographic variables (age class, ethnic background), socioeconomic variables (socioeconomic status, education), and lifestyle variables (alcohol intake frequency, smoking history, body mass index, duration of walks, sleep duration, blood pressure, medication intake).

### Sensitivity analysis

In the UKB cohort, linear relationships were observed between the four blood biomarkers mentioned before and the incidence of BPH (**Table 3**). When all the demographic variables, socioeconomic variables and lifestyle variables were included in the analysis, the results were similar to those obtained in the main analyses as reported in model III (**Table 2**). The HDL (HR_Q4 vs. Q1_ = 0.87; 95% CI = 0.83–0.91), apolipoprotein A (HR_Q4 vs. Q1_ = 0.86; 95% CI = 0.83–0.90) and gamma glutamyltransferase (HR_Q4 vs. Q1_ = 0.93; 95% CI = 0.88–0.97) demonstrate a protective effect against the incidence of BPH, while IGF-1 (HR_Q4 vs. Q1_ = 1.07; 95% CI = 1.03–1.12) on the contrary. Nevertheless, the associations were weakened upon adjustment for additional covariates.

**Table 3:** Hazard ratios and 95% confidence interval for the association between quartile of blood biomarker levels and the risk of BPH in the UKB cohort

Range	Cases	Median	Model I*		Model II†		Model III‡	
			**HR**	**95% CI**	**HR**	**95% CI**	**HR**	**95% CI**
**High-density lipoprotein cholesterol (mmol/L)**								
0.252–1.066	37 355	0.958	Ref		Ref		Ref	
1.067–1.242	37 211	1.156	0.94	0.90–0.98	0.95	0.91–0.99	0.98	0.94–1.03
1.243–1.456	37 082	1.339	0.89	0.85–0.92	0.89	0.85–0.92	0.94	0.90–0.98
1.457–3.418	37 133	1.637	0.81	0.78-0.85	0.79	0.76–0.83	0.87	0.83–0.91
***P*-value for trend**			<0.001		<0.001		<0.001	
**Apolipoprotein A (mmol/L)**								
0.419–1.274	37 363	1.187	Ref		Ref		Ref	
1.275–1.412	37 246	1.346	0.96	0.92–0.99	0.94	0.91–0.98	0.97	0.93–1.01
1.413–1.567	37 035	1.482	0.89	0.85–0.92	0.86	0.82–0.89	0.90	0.87–0.94
1.568–2.498	37 137	1.694	0.85	0.82–0.89	0.80	0.76–0.83	0.86	0.83–0.90
***P*-value for trend**			<0.001		<0.001		<0.001	
**Insulin-like growth factor-1**								
2.019–18.068	37 201	15.626	Ref		Ref		Ref	
18.069–21.557	37 193	19.932	0.98	0.94–1.02	1.05	1.01–1.09	1.06	1.02–1.10
21.558–24.890	37 192	23.121	0.94	0.90–0.98	1.05	1.01–1.10	1.06	1.01–1.10
24.891–126.766	37 195	27.489	0.91	0.87–0.95	1.08	1.03–1.12	1.07	1.03–1.12
***P*-value for trend**			<0.001		<0.001		0.002	
**Gamma glutamyltransferase**								
6.2–24.0	37 507	19.700	Ref		Ref		Ref	
24.1–33.5	37 017	28.400	0.99	0.95–1.03	0.97	0.93–1.01	0.96	0.92–1.00
33.6–50.8	37 112	40.400	1.01	0.97–1.05	0.99	0.95–1.03	0.98	0.94–1.02
50.9–1184.9	37 145	72.600	0.92	0.88–0.96	0.93	0.89–0.97	0.93	0.88–0.97
***P*-value for trend**			<0.001		0.002		0.002	

BPH – benign prostatic hyperplasia, CI – confidence interval, HR – hazard ratio, Ref – reference, UKB – UK Biobank

*Model I adjusted for non.

†Model II adjusted for demographic (age class, ethnic background) and socioeconomic variables (socioeconomic status, education).

‡Model III adjusted for demographic (age class, ethnic background), socioeconomic (socioeconomic status, education), and lifestyle variables (alcohol intake frequency, smoking history, body mass index, duration of walks, sleep duration, blood pressure, medication intake).

### Subgroup analysis

In the UKB cohort, based on the results of the main analysis, we performed subgroup analyses for HDL, apolipoprotein A in the model III analysis (adjusting for demographic variables, socioeconomic variables and lifestyle variables), In the subgroup analyses, most of the results (age class, Townsend, qualifications, smoking history, alcohol intake frequency, BMI, duration of walks, sleep duration, blood pressure) closely paralleled those observed in the entire cohort as shown in Tables S3–S4 in the **Online Supplementary Document**, albeit with a few exceptions. Specifically, as for apolipoprotein A, male participants who take medication like Cholesterol lowering medication (HR = 0.91; 95% CI = 0.75–1.11, *P* = 0.355) and insulin (HR = 2.12; 95% CI = 0.59–7.63, *P* = 0.252) were observed with a nonsignificant association. In Asian/Asian British (HR = 0.44; 95% CI = 0.29–0.67, *P* < 0.001) and White (HR = 0.78; 95% CI = 0.74–0.82, *P* < 0.001) ethnic background subgroups, male participants were observed with a protective effect of HDL on prostatic hyperplasia, which was not significant in Black/Black British, mixed and other ethnic group.

### Validation in the CHALRS cohort

As for CHARLS cohort, the baseline characteristics of the participants along with venous blood-based biomarkers, are presented in Table S2 in the **Online Supplementary Document**. Concurrently, age discrepancy was observed between the BPH-afflicted cohort and the non-BPH cohort. Moreover, statistically significant disparities were observed in residency status, educational attainment, prevalence of hypertension, lung diseases, stroke, liver diseases, kidney diseases, stomach/digestive disorders, and asthma between the cohort exhibiting LUTS/BPH symptoms and the asymptomatic group.

In the CHARLS cohort, the results of multivariate logistic regression analyses at the cross-sectional level are presented in **Table 4**. The unadjusted model unveiled a notable association between venous blood-based biomarkers and the probability of BPH development. Specifically, it demonstrated that participants exhibiting elevated levels of creatinine, glucose, cystatin C, and glycated haemoglobin are predisposed to an elevated risk of BPH development. Conversely, individuals with elevated levels of HDL were associated with a decreased risk of BPH development. After controlling for all potential variables, individuals with elevated high-density lipoprotein cholesterol (OR = 0.992; 95% CI = 0.985–0.999, *P* = 0.033) were found to be associated with a decreased risk of developing BPH. It can be observed from all four models that there is a significantly negative correlation between high-density lipoprotein cholesterol and benign prostatic hyperplasia (**Table 4**).

**Table 4:** Multivariate logistic regression identify the association between venous blood-based biomarkers and BPH in the CHARLS cohort

Biomarkers	OR	95%CI	*P*-value
Crude model*			
White blood cell	0.97	(0.93–1.01)	0.11
Haemoglobin	1.01	(0.97–1.05)	0.71
Haematocrit	1.01	(0.99–1.02)	0.24
Mean corpuscular volume	1.00	(0.99–1.01)	0.91
Platelets	1.00	(1.00–1.00)	0.69
Triglycerides	1.00	(1.00–1.00)	0.92
Creatinine	1.33	(1.08–1.63)	0.01
Blood urea nitrogen	1.01	(0.99–1.02)	0.48
High-density lipoprotein cholesterol	0.99	(0.98–1.00)	0.00
Low-density lipoprotein cholesterol	1.00	(1.00–1.00)	0.50
Total cholesterol	1.00	(1.00–1.00)	0.84
Glucose	1.00	(1.00–1.00)	0.02
Uric acid	1.04	(0.98–1.09)	0.19
Cystatin C	1.68	(1.28–2.19)	0.00
C-reactive protein	1.01	(1.00–1.02)	0.19
Glycated haemoglobin	1.09	(1.01–1.17)	0.03
**Model I†**			
White blood cell	0.98	(0.94–1.02)	0.35
Haemoglobin	1.07	(1.02–1.12)	0.00
Haematocrit	1.02	(1.01–1.04)	0.00
Mean corpuscular volume	1.00	(0.99–1.01)	0.38
Platelets	1.00	(1.00–1.00)	0.58
Triglycerides	1.00	(1.00–1.00)	0.05
Creatinine	1.17	(0.96–1.43)	0.11
Blood urea nitrogen	1.00	(0.98–1.01)	0.56
High-density lipoprotein cholesterol	0.99	(0.98–1.00)	0.00
Low-density lipoprotein cholesterol	1.00	(1.00–1.00)	0.49
Total cholesterol	1.00	(1.00–1.00)	0.67
Glucose	1.00	(1.00–1.00)	0.02
Uric acid	1.04	(0.98–1.10)	0.17
Cystatin C	1.06	(0.78–1.43)	0.72
C-reactive protein	1.00	(0.99–1.01)	0.54
Glycated haemoglobin	1.07	(0.99–1.16)	0.08
**Model II‡**			
White blood cell	0.98	(0.94–1.02)	0.35
Haemoglobin	1.06	(1.01–1.10)	0.02
Haematocrit	1.02	(1.01–1.04)	0.01
Mean corpuscular volume	1.00	(0.99–1.01)	0.31
Platelets	1.00	(1.00–1.00)	0.56
Triglycerides	1.00	(1.00–1.00)	0.23
Creatinine	1.13	(0.92–1.39)	0.26
Blood urea nitrogen	1.00	(0.98–1.01)	0.82
High-density lipoprotein cholesterol	0.99	(0.99–1.00)	0.02
Low-density lipoprotein cholesterol	1.00	(1.00–1.00)	0.79
Total cholesterol	1.00	(1.00–1.00)	0.88
Glucose	1.00	(1.00–1.00)	0.12
Uric acid	1.02	(0.96–1.07)	0.59
Cystatin C	0.99	(0.72–1.36)	0.95
C-reactive protein	1.01	(1.00–1.01)	0.36
Glycated haemoglobin	1.04	(0.96–1.13)	0.29
**Model III§**			
White blood cell	0.98	(0.94–1.02)	0.32
Haemoglobin	1.05	(1.01–1.10)	0.03
Haematocrit	1.02	(1.01–1.04)	0.01
Mean corpuscular volume	1.00	(0.99–1.01)	0.35
Platelets	1.00	(1.00–1.00)	0.55
Triglycerides	1.00	(1.00–1.00)	0.36
Creatinine	0.96	(0.76–1.21)	0.73
Blood urea nitrogen	0.99	(0.98–1.01)	0.40
High-density lipoprotein cholesterol	0.99	(0.99–1.00)	0.03
Low-density lipoprotein cholesterol	1.00	(1.00–1.00)	0.84
Total cholesterol	1.00	(1.00–1.00)	0.91
Glucose	1.00	(1.00–1.00)	0.07
Uric acid	1.00	(0.95–1.06)	0.92
Cystatin C	0.75	(0.53–1.04)	0.09
C-reactive protein	1.00	(0.99–1.01)	0.64
Glycated haemoglobin	1.03	(0.95–1.12)	0.42

CI – confidence interval, OR – odds ratio, Q – quartile

*Crude model adjusted for none.

†Model I adjusted for age.

‡Model II adjusted for age, education, residence, marital status, smoking, and drinking condition.

§Model III adjusted for age, education, residence, marital status, smoking condition, drinking condition, hypertension, lung diseases, stroke, psych problems, arthritis, liver diseases, kidney diseases, stomach/digestive diseases, and asthma.

## DISCUSSION

As one of the most prevalent chronic diseases affecting aging males, the burden of BPH is growing over the world [18]. Our study revealed that substantial changes in some blood biomarkers are significantly associated with the incidence of BPH among middle-aged and older men in the UK biobank and CHARLS cohort. In this large prospective cohort study comprising two sizable cohorts from Europe and East Asia, we examined the correlation between biomarkers indicative of blood cell count, liver function, lipid metabolism, renal function, hormone levels, and blood glucose regulation, and the propensity for developing BPH. The findings revealed an inverse dose-response correlation between apolipoprotein A and HDL levels, with the risk of BPH following adjustments for all pertinent demographic, socioeconomic, and lifestyle variables

Research investigating the correlation between lipid metabolism and the risk of BPH have drawn conflicting results, especially the HDL, showing inverse and positive associations depending upon study design in the previous studies [4,19,20]. High-density lipoprotein fulfils a pivotal function in reverse cholesterol transport, the process responsible for removing surplus cholesterol from the cells and tissues of the body, subsequently conveying it back to the liver for excretion. In the meantime, apolipoprotein A is the primary protein component of high-density lipoprotein. Besiroglu [19] found that TG, HDL, and TG/HDL levels were found to be associated with prostate enlargement in a cross-sectional study including 400 Turkish men with BPH symptoms. A 5-year follow up study of Martin [20], including 780 male participants aged from 35 to 80 years, revealed that participants with elevated levels of HDL were prone to experiencing improvements in the storage of lower urinary tract symptoms. On the contrary, a prospective cohort investigation conducted by Parsons [4] found that there were no overall associations between BPH and lipids and lipoproteins.

In this prospective cohort study, we identified a nonlinear association between HDL/apolipoprotein A and the risk of BPH after adjusting for all potential covariates. This correlation could potentially be attributed to fluctuations in hormone levels and systemic inflammation. The study of Upreti [21] found that HDL may play a role in BPH development by regulating hormone levels such as dihydrotestosterone, which is believed to be key factors in BPH [22]. Also, HDL can increase the production of SHBG, which can bind to free testosterone and DHT, reducing their activity in prostate tissue and thus decreasing the risk of BPH [21]. In addition, the study of Zhang [23] indicate that simvastatin and atorvastatin substantially decreased prostate volume, ameliorated symptoms of the lower urinary tract, and delayed the clinical progression of BPH, presumably by lowering cholesterol and anti-inflammatory factors. Systemic inflammation is another crucial potential mechanism. According to a retrospective study involving 271 members treated with simple prostatectomy for BPH [24], prostate volume was significantly associated with dyslipidaemia including HDL, which helps to remove LDL and cholesterol from the body and has antioxidant and anti-inflammatory properties. Several studies have indicated a potential association between hypertriglyceridemia and reduced levels of HDL with prostatic inflammation mediated by the interleukin-8 (IL-8) response. These findings suggest that these factors could exacerbate inflammation and tissue remodelling in individuals suffering from BPH or LUTS [25]. Substances associated with pervasive inflammation have the capacity to induce the proliferation of both epithelial and stromal cells within the prostate, thereby contributing to the pathogenesis of BPH [26]. Therefore, systemic inflammation, functioning as a mediator in the association between HDL levels and BPH, may plausibly contribute to the increased prevalence rate of BPH.

Also, IGF-1 (HR = 1.00; 95% CI = 1.00–1.01, *P* < 0.001) and gamma glutamyl transferase (HR = 0.99; 95% CI = 0.99–1.00, *P* < 0.001) remained highly correlated with incident BPH after controlling for all potential confounders. A study investigating antagonists of growth hormone-releasing hormone (GHRH) has demonstrated their potential to reduce prostate weight in experimental BPH animal models. The inhibitory effect of these GHRH analogues is partially mediated through indirect endocrine mechanisms, wherein they suppress GHRH-induced release of growth hormone (GH) from the pituitary gland. This suppression subsequently leads to the inhibition of hepatic production of IGF-1, which suggesting that the elevated levels of IGF may cause hypertrophy of the prostate and lead to increased morbidity [27].

Benign prostatic hyperplasia is widely recognised as a chronic hyperplastic process primarily influenced androgen stimuli. However, the incidence of BPH escalates with age coinciding with a decline in blood testosterone levels associated with aging, suggesting the potential involvement of additional local paracrine or mitogenic factors in the regulation of prostate enlargement. While epidemiological data regarding the association between serum androgen concentrations and BPH exhibit inconsistency, there is a prevailing consensus that androgens assume a permissive role in the pathogenesis of BPH [28]. In plasma, the majority of circulating steroid hormones are bound to carrier proteins. The principal binding proteins include albumin, SHBG, and cortisol-binding globulin. Numerous studies have investigated potential associations between the risk of BPH and circulating levels of androgens, such as testosterone and dihydrotestosterone (DHT), but a consistent relationship has not been conclusively established yet [29,30]. Rohrmann [30] reported that there is no significant differences in testosterone levels, free testosterone, or SHBG between control participants and men with LUTS in a study of NHANES III data. Conversely, elevated levels of total and bioavailable testosterone were found to be correlated with a decreased risk of BPH in a clinical trial focused on prostate cancer prevention [31]. From the results of our cohort study, we believe that the plasma testosterone level, or SHBG level may not affect the development of prostatic hyperplasia because plasma levels of androgens may not accurately reflect their concentrations within the prostate gland. The prostate gland is characterised by an abundance of androgen-metabolising enzymes, notably 5α-reductase, catalysing the conversion of testosterone to the more potent dihydrotestosterone (DHT), which lead to the hypothesis that the local accumulation of DHT within the prostate may contribute to the pathogenesis of BPH [32].

In previous studies, serum glucose and HbA1c were often included as part of Metabolic Syndrome to explore the relationship of BPH and elevated glucose and glycated haemoglobin levels tended to increase the risk of BPH [8,33,34]. However, the sample size of several hundred individuals and the cross-sectional study design cannot provide accurate prediction of the association between glycaemic parameters and BPH risk. To our knowledge, this study is the largest cohort studies of glycaemic parameters and BPH risk to date. In our cohort study, there were statistically significant differences in blood glucose related indicators between patients and controls at Baseline level. However, there was no association between blood glucose and BPH in either the UKB or CHARLS cohort study with adjustment for covariates. Additionally, our study found no significant associations between prostatic hyperplasia pathogenesis and various indicators, including inflammatory markers, vitamin and electrolyte levels, renal function, and blood cell counts.

Our study identifies a correlation between higher levels of HDL and apolipoprotein A and a reduced incidence of BPH in a large cohort of middle-aged and elderly individuals. These findings suggest that public health initiatives promoting lifestyle modifications to improve HDL and apolipoprotein A levels – such as increasing physical activity, adopting healthy diets rich in unsaturated fats, and smoking cessation – could potentially lower the risk of BPH. Additionally, routine screening for these biomarkers in at-risk populations may offer valuable insights for early intervention strategies. Future research should include randomised controlled trials to evaluate the efficacy of these interventions in individuals with BPH.

While this research was based on a broad cohort of European and East Asian populations, it did encounter several limitations. First, the diagnostic methods employed to assess BPH within the UK Biobank and CHARLS cohorts exhibited inconsistencies, each method presenting certain limitations. Benign prostatic hyperplaisa diagnosis of the UKB cohort relied on inpatient records and death registries from Hospital Episode Statistics for England (HES), Scottish Morbidity Record (SMR) and Patient Episode Database for Wales (PEDW). In the CHARLS cohort, the diagnosis of BPH was primarily based on self-reported responses in questionnaires, lacking validation through cross-referencing with medical records. The UK Biobank primarily includes participants from the European, who may have different lifestyle factors, genetic backgrounds, and environmental exposures compared to the Chinese participants in CHARLS. These demographic and health care system differences may introduce biases. Second, the BPH diagnosis questionnaire in the CHARLS database was administered biennially. Consequently, we utilised only CHARLS Wave 2, which included blood biomarker data, for the cross-sectional analysis. Additionally, potential bias may arise from the different methods used to measure blood biomarkers between the two cohorts. Third, a significant difference in sample sizes existed between the two cohorts. The UKB cohort comprised over 200 000 male participants, while the CHARLS cohort consisted of 20 284 participants. Despite observing associations between HDL and BPH in both cohorts, the strength of evidence within the Asian population remains relatively constrained, and the incorporation of blood biomarkers was less comprehensive compared to the UKB cohort. Future research should encompass larger-scale Asian cohorts and incorporate a broader array of blood biomarkers. Fourthly, due to differences in study design, there were differences in the covariates included in the CHALRS and UKB cohort study, and residual confounding may persist despite our efforts to control for potential confounding variables. Finally, the lack of randomised interventions poses limitations on the robustness of evidence for establishing causal relationships.

## CONCLUSIONS

This large prospective biomarker-based study focused on risk of developing BPH, which has not been further investigated in previous studies on large population cohort. Therefore, our research may contribute to knowledge on the relationship between circulating levels of blood biomarkers and progression of BPH. Our findings suggest that HDL and apolipoprotein A are significant protective factors in progress of the BPH and biomarkers such as glucose-related biomarkers, inflammatory markers, and hormone levels do not play a significant role in the progression of BPH. In conjunction with previous data from experimental and observational studies, our study provides support to the potential involvement of the lipid biomarkers in the early stages of BPH development and future strategies should prioritise lipid-related pathways in the prevention and management of BPH. Future research should include randomised controlled trials to evaluate the efficacy of these interventions in individuals with BPH.


Online Supplementary Document

