# Supplementary material for: Circulating levels of blood biomarkers and risk of benign prostatic hyperplasia: Results from two large cohorts in Europe and East Asia
Source: J Glob Health. 2024 Nov 22;14:04242. doi: 10.7189/jogh.14.04242 (PMC11586646; doi:10.7189/jogh.14.04242)
Supplement: Online Supplementary Document [file jogh-14-04242-s001.pdf]

Supplementary Material

Circulating levels of blood biomarkers and risk of benign prostatic hyperplasia: results from two large cohorts in Europe and East Asia

Shengzhuo Liu[1#] Xiaoyang Liu[1#] Pan Song[1] Luchen Yang[1] Zhenghuan Liu[1] Jing Zhou[1] Linchun Wang[1] Xin Yan[1] Kai Ma[1] Yunfei Yu[1] Xianding Wang[1,2,*] Qiang Dong[1,*]

[1] Department of Urology, Institute of Urology, West China Hospital, Sichuan University, Chengdu, China

[2] Kidney Transplantation Center, West China Hospital, Sichuan University, Chengdu, China

*** Correspondence:**
Xianding Wang; Qiang Dong
E-mail: xiandingwang@scu.edu.cn (WX). dongqiang@scu.edu.cn (DQ)

Table S1. Baselines characteristics of the participants' blood biomarkers level in the UK biobank(SD: standard deviation, SD for continuous variables: P value was calculated by Kruskal Wallis rank-sum test, Number (%) for categorical variables: P value was calculated by chi-square test BPH: benign prostatic hyperplasia

		BPH			
	**Category/Unit**	**Yes**	**No**	**P**	**Distribution**
		**n=17633**	**n=131148**		
Basophill count (median [IQR])	10^9 cells/Litre	0.02 [0.00, 0.04]	0.02 [0.00, 0.04]	0.009	nonnorm
Eosinophill count (median [IQR])	10^9 cells/Litre	0.16 [0.10, 0.24]	0.16 [0.10, 0.24]	0.088	nonnorm
Haematocrit percentage (median [IQR])	percent	43.36 [41.48, 45.22]	43.38 [41.40, 45.31]	0.259	nonnorm
Haemoglobin concentration (median [IQR])	grams/decilitre	15.00 [14.39, 15.66]	15.01 [14.37, 15.68]	0.941	nonnorm
Lymphocyte count (median [IQR])	10^9 cells/Litre	1.80 [1.48, 2.20]	1.80 [1.44, 2.20]	<0.001	nonnorm
Mean corpuscular haemoglobin (median [IQR])	picograms	31.72 [30.75, 32.73]	31.66 [30.70, 32.66]	<0.001	nonnorm
Mean corpuscular volume (median [IQR])	femtolitres	91.60 [88.99, 94.25]	91.41 [88.84, 94.09]	<0.001	nonnorm
Mean platelet thrombocyte volume (median [IQR])	femtolitres	9.16 [8.52, 9.90]	9.17 [8.50, 9.90]	0.74	nonnorm
Mean reticulocyte volume (median [IQR])	femtolitres	106.40 [101.92, 111.10]	106.30 [101.90, 110.93]	0.094	nonnorm
Monocyte count (median [IQR])	10^9 cells/Litre	0.50 [0.40, 0.60]	0.50 [0.40, 0.61]	<0.001	nonnorm
Neutrophill count (median [IQR])	10^9 cells/Litre	4.09 [3.30, 5.00]	4.19 [3.40, 5.10]	<0.001	nonnorm
Nucleated red blood cell count (median [IQR])	10^9 cells/Litre	0.00 [0.00, 0.00]	0.00 [0.00, 0.00]	0.428	nonnorm
Platelet count (median [IQR])	10^9 cells/Litre	233.90 [201.90, 269.20]	232.00 [199.80, 268.00]	<0.001	nonnorm
Red blood cell erythrocyte count (median [IQR])	10^12 cells/Litre	4.74 [4.50, 4.98]	4.75 [4.51, 4.99]	<0.001	nonnorm
Reticulocyte count (median [IQR])	10^12 cells/Litre	0.06 [0.05, 0.08]	0.06 [0.05, 0.08]	0.296	nonnorm
White blood cell leukocyte count (median [IQR])	10^9 cells/Litre	6.70 [5.70, 7.90]	6.80 [5.78, 8.00]	<0.001	nonnorm
Alanine aminotransferase (median [IQR])	U/L	23.84 [18.48, 31.80]	23.26 [18.16, 30.75]	<0.001	nonnorm
Albumin (median [IQR])	g/L	45.46 [43.78, 47.14]	45.10 [43.44, 46.79]	<0.001	nonnorm
Alkaline phosphatase (median [IQR])	U/L	79.00 [67.10, 93.10]	79.40 [67.50, 94.00]	<0.001	nonnorm
Apolipoprotein A (median [IQR])	g/L	1.41 [1.28, 1.57]	1.40 [1.26, 1.55]	<0.001	nonnorm

Apolipoprotein B (median [IQR])	g/L	1.02 [0.86, 1.19]	0.99 [0.84, 1.16]	<0.001	nonnorm
Aspartate aminotransferase (median [IQR])	U/L	26.20 [22.70, 31.00]	25.90 [22.40, 30.50]	<0.001	nonnorm
C reactive protein (median [IQR])	mg/L	1.30 [0.68, 2.57]	1.37 [0.71, 2.69]	<0.001	nonnorm
Calcium (median [IQR])	mmol/L	2.37 [2.31, 2.43]	2.36 [2.31, 2.42]	<0.001	nonnorm
Cholesterol (median [IQR])	mmol/L	5.49 [4.73, 6.25]	5.32 [4.55, 6.09]	<0.001	nonnorm
Creatinine (median [IQR])	umol/L	80.00 [72.60, 88.40]	80.50 [72.90, 89.10]	<0.001	nonnorm
Cystatin C (median [IQR])	mg/L	0.92 [0.84, 1.01]	0.94 [0.86, 1.04]	<0.001	nonnorm
Direct bilirubin (median [IQR])	umol/L	1.79 [1.43, 2.30]	1.81 [1.46, 2.32]	<0.001	nonnorm
Gamma glutamyltransferase (median [IQR])	U/L	33.60 [24.00, 51.00]	33.20 [23.90, 49.30]	0.001	nonnorm
Glucose (median [IQR])	mmol/L	4.96 [4.62, 5.37]	4.98 [4.62, 5.41]	<0.001	nonnorm
HbA1c (median [IQR])	mmol/mol	35.40 [32.90, 38.20]	35.80 [33.30, 38.80]	<0.001	nonnorm
HDL cholesterol (median [IQR])	mmol/L	1.25 [1.07, 1.46]	1.22 [1.05, 1.43]	<0.001	nonnorm
IGF 1 (median [IQR])	nmol/L	21.59 [18.09, 24.92]	21.36 [17.87, 24.70]	<0.001	nonnorm
LDL direct (median [IQR])	mmol/L	3.48 [2.88, 4.07]	3.36 [2.76, 3.96]	<0.001	nonnorm
Lipoprotein A (median [IQR])	nmol/L	19.65 [9.16, 61.01]	20.10 [9.20, 61.20]	0.403	nonnorm
Phosphate (median [IQR])	mmol/L	1.12 [1.01, 1.23]	1.11 [1.01, 1.22]	0.016	nonnorm
SHBG (median [IQR])	nmol/L	37.41 [28.44, 48.66]	38.55 [29.54, 49.76]	<0.001	nonnorm
Testosterone (median [IQR])	nmol/L	11.56 [9.38, 14.05]	11.48 [9.29, 13.89]	<0.001	nonnorm
Total bilirubin (median [IQR])	umol/L	9.11 [7.34, 11.64]	9.19 [7.41, 11.66]	0.012	nonnorm
Total protein (median [IQR])	g/L	72.39 [69.86, 75.09]	71.96 [69.40, 74.68]	<0.001	nonnorm
Triglycerides (median [IQR])	mmol/L	1.70 [1.19, 2.46]	1.71 [1.20, 2.40]	0.461	nonnorm
Urate (median [IQR])	umol/L	350.70 [306.20, 399.70]	348.80 [303.70, 398.90]	0.001	nonnorm
Urea (median [IQR])	mmol/L	5.48 [4.70, 6.35]	5.63 [4.84, 6.53]	<0.001	nonnorm
Vitamin D (median [IQR])	nmol/L	46.80 [32.50, 62.10]	48.60 [33.90, 63.90]	<0.001	nonnorm

(Median and IQR are used to describe continuous data with a skewed distribution. Rates and percentages are used to represent categorical variables. The K-W test was used to calculate p-values for continuous variables

with skewed distributions, while chi-square tests were used to assess categorical data. Abbreviations: HbA1c, glycated hemoglobin; HDL, high-density lipoprotein cholesterol; IGF-1, insulin-like growth factor-1; SHBG, Sex Hormone Binding Globulin)

Table S2. Baselines characteristics of the participants enrolled (SD: standard deviation, SD for continuous variables: P value was calculated by Kruskal Wallis rank-sum test, Number (%) for categorical variables: P value was calculated by chi-square test BPH: benign prostatic hyperplasia

		BPH		
	Definition	Yes	No	P
	Category/Unit	n=746	n=5081	
Age class (%)	<50 year	60 (8.04)	685 (13.48)	<0.001
	<60 year	156 (20.91)	1705 (33.56)	
	<70 year	290 (38.87)	1779 (35.01)	
	>=70 year	240 (32.17)	912 (17.95)	
residence (%)	urban	238 (35.63)	916 (20.14)	<0.001
	rural	430 (64.37)	3632 (79.86)	
Education level (%)	Less than lower secondary	596 (79.89)	4400 (86.61)	<0.001
	Upper secondary & vocational training	114 (15.28)	592 (11.65)	
	tertiary	36 (4.83)	88 (1.73)	
Marital status (%)	married or partnered	658 (88.20)	4584 (90.22)	0.1
	separated divorced widowed or never married	88 (11.80)	497 (9.78)	
Smoking (%)	Yes	595 (79.76)	4207 (82.83)	0.045
	No	151 (20.24)	872 (17.17)	
Drinking (%)	Yes	531 (71.28)	3724 (73.42)	0.234
	No	214 (28.72)	1348 (26.58)	
Hypertension (%)	no	351 (53.51)	2958 (67.46)	<0.001
	yes	305 (46.49)	1427 (32.54)	
Lung diseases (%)	no	540 (83.33)	4028 (92.07)	<0.001
	yes	108 (16.67)	347 (7.93)	
Stroke (%)	no	478 (73.20)	3705 (84.03)	<0.001
	yes	175 (26.80)	704 (15.97)	
Psych problem (%)	no	448 (68.61)	3774 (85.95)	<0.001
	yes	205 (31.39)	617 (14.05)	
Arthritis (%)	no	597 (92.27)	4244 (96.08)	<0.001
	yes	50 (7.73)	173 (3.92)	
Liver diseases (%)	no	633 (96.94)	4351 (98.57)	0.004
	yes	20 (3.06)	63 (1.43)	
Kidney diseases (%)	no	358 (54.66)	2730 (61.72)	0.001
	yes	297 (45.34)	1693 (38.28)	

Stomach/digestive diseases (%)	no	429 (67.03)	3566 (82.89)	<0.001
	yes	211 (32.97)	736 (17.11)	
Asthma (%)	no	549 (84.98)	4115 (93.65)	<0.001
	yes	97 (15.02)	279 (6.35)	
White Blood Cell	10^9/L	5.80 [4.84, 7.10]	5.96 [5.00, 7.12]	0.039
Hemoglobin	g/dl	14.60 [13.50, 15.50]	14.60 [13.60, 15.60]	0.52
Hematocrit	%	43.80 [40.70, 46.70]	43.90 [40.80, 46.90]	0.871
Mean Corpuscular Volume	fl	93.00 [89.43, 96.77]	93.10 [89.20, 96.90]	0.771
Platelets	10^9/L	187.00 [151.25, 226.00]	192.00 [153.00, 231.00]	0.112
Triglycerides	mg/dl	108.41 [77.88, 161.50]	107.08 [77.88, 163.72]	0.861
Creatinine	mg/dl	0.89 [0.79, 1.01]	0.87 [0.77, 0.98]	<0.001
Blood Urea Nitrogen (BUN)	mg/dl	15.41 [12.89, 18.77]	15.69 [12.89, 18.77]	0.846
High-density Lipoprotein Cholesterol	mg/dl	46.91 [40.15, 54.44]	48.65 [41.31, 56.76]	0.001
Low Density Lipoprotein Cholesterol	mg/dl	97.68 [79.92, 116.60]	96.53 [79.54, 114.67]	0.386
Total Cholesterol	mg/dl	173.55 [153.67, 198.75]	174.90 [154.05, 197.68]	0.843
Glucose	mg/dl	97.30 [90.09, 108.11]	95.50 [88.29, 106.31]	0.003
Uric Acid	mg/dl	5.50 [4.52, 6.40]	5.40 [4.60, 6.30]	0.125
Cystatin C	mg/l	0.89 [0.78, 1.01]	0.85 [0.75, 0.97]	<0.001
C-Reactive Protein	mg/l	1.50 [0.80, 2.80]	1.40 [0.80, 2.70]	0.26
Glycated Hemoglobin	%	5.80 [5.50, 6.20]	5.80 [5.50, 6.10]	0.01

Table S3. Subgroup analysis between HDL and BPH; HR Hazard ratios; 95% CI: 95% Confidence interval;

Variable	Count	Percent	HR	Lower	Upper	P value	P for interaction
age class							0.470
<50	22598	15.2	0.78	0.62	0.98	0.035	
<60	55110	37	0.71	0.64	0.78	<0.001	
>=60	71073	47.8	0.75	0.71	0.8	<0.001	
Townsend							0.270
<=-3.96	30422	20.4	0.81	0.72	0.9	<0.001	
<=-2.81	30189	20.3	0.76	0.69	0.85	<0.001	
<=-1.34	30050	20.2	0.69	0.62	0.77	<0.001	
<=1.35	29612	19.9	0.8	0.72	0.9	<0.001	
>1.35	28508	19.2	0.79	0.7	0.88	<0.001	
Qualifications							0.936
A levels/AS levels or equivalent	15421	10.4	0.81	0.69	0.95	0.011	
College or University degree	49811	33.5	0.77	0.71	0.84	<0.001	
CSEs or equivalent	7479	5	0.8	0.6	1.06	0.117	
None of the above	27236	18.3	0.81	0.73	0.89	<0.001	
NVQ or HND or HNC or equivalent	14207	9.5	0.72	0.61	0.84	<0.001	
O levels/GCSEs or equivalent	27572	18.5	0.79	0.7	0.89	<0.001	
Other professio l qualifications eg: nursing, teaching	7055	4.7	0.81	0.65	1	0.053	
Ethnic background							0.024
Asian or Asian British	3145	2.1	0.44	0.29	0.67	<0.001	
Black or Black British	1535	1	0.63	0.39	1.04	0.072	
Mixed	582	0.4	0.84	0.38	1.84	0.657	
Other ethnic group	984	0.7	1.32	0.75	2.34	0.337	
White	142535	95.8	0.78	0.74	0.82	<0.001	
Smoking history							0.893
No	71467	48	0.78	0.72	0.84	<0.001	
Yes	77314	52	0.77	0.72	0.82	<0.001	
Alcohol intake frequency							0.054
None	8732	5.9	1	0.81	1.23	0.982	
<3	60714	40.8	0.76	0.7	0.83	<0.001	
>=3	79335	53.3	0.82	0.77	0.88	<0.001	
BMI							0.524
underweight	353	0.2	1.06	0.47	2.41	0.884	
normal	36283	24.4	0.82	0.75	0.9	<0.001	
overweight	73799	49.6	0.78	0.72	0.84	<0.001	
Obesity	38346	25.8	0.75	0.66	0.84	<0.001	
Duration of walks							0.636
<15	12583	8.5	0.74	0.62	0.88	<0.001	
<30	36694	24.7	0.74	0.67	0.82	<0.001	
<60	43403	29.2	0.76	0.69	0.83	<0.001	
<180	42821	28.8	0.81	0.74	0.89	<0.001	
>=180	13280	8.9	0.81	0.69	0.96	0.017	
Sleep duration							0.545
<7	36807	24.7	0.77	0.7	0.85	<0.001	

<10	109335	73.5	0.78	0.74	0.83	<0.001	
>=10	2639	1.8	0.65	0.46	0.9	0.01	
Blood pressure							0.066
normal	15739	10.6	0.69	0.59	0.8	<0.001	
High-normal	15646	10.5	0.64	0.55	0.75	<0.001	
Grade 1	38693	26	0.79	0.71	0.88	<0.001	
Grade 2	74225	49.9	0.8	0.75	0.86	<0.001	
Grade 3	4478	3	0.78	0.6	1.01	0.055	
Medication							0.257
Blood pressure medication	15538	10.4	0.79	0.69	0.91	0.001	
Cholesterol lowering medication	12674	8.5	0.92	0.79	1.08	0.3	
Insulin	266	0.2	1.37	0.56	3.38	0.494	
Mixed	24022	16.1	0.8	0.71	0.89	<0.001	
None of the above	96281	64.7	0.88	0.82	0.94	<0.001	

Table S4. Subgroup analysis between Apolipoprotein A and BPH; HR Hazard ratios; 95% CI: 95% Confidence interval;

Variable	Count	Percent	HR	Lower	Upper	P value	P for interaction
age class							0.420
<50	22598	15.2	0.77	0.57	1.04	0.086	
<60	55110	37	0.62	0.55	0.71	<0.001	
>=60	71073	47.8	0.67	0.61	0.72	<0.001	
Townsend							0.254
<=-3.96	30422	20.4	0.75	0.65	0.87	<0.001	
<=-2.81	30189	20.3	0.71	0.61	0.82	<0.001	
<=-1.34	30050	20.2	0.66	0.57	0.76	<0.001	
<=1.35	29612	19.9	0.81	0.7	0.94	0.006	
>1.35	28508	19.2	0.79	0.68	0.91	0.001	
Qualifications							0.970
A levels/AS levels or equivalent	15421	10.4	0.76	0.61	0.94	0.011	
College or University degree	49811	33.5	0.73	0.65	0.82	<0.001	
CSEs or equivalent	7479	5	0.73	0.5	1.06	0.096	
None of the above	27236	18.3	0.77	0.67	0.88	<0.001	
NVQ or HND or HNC or equivalent	14207	9.5	0.71	0.57	0.87	0.001	
O levels/GCSEs or equivalent	27572	18.5	0.79	0.68	0.92	0.003	
Other professiol qualifications eg: nursing, teaching	7055	4.7	0.78	0.59	1.04	0.095	
Ethnic background							0.219
Asian or Asian British	3145	2.1	0.46	0.28	0.76	0.003	
Black or Black British	1535	1	0.61	0.32	1.18	0.141	
Mixed	582	0.4	0.92	0.32	2.62	0.88	
Other ethnic group	984	0.7	1.26	0.57	2.77	0.564	
White	142535	95.8	0.75	0.7	0.8	<0.001	
Smoking history							0.523
No	71467	48	0.76	0.69	0.84	<0.001	
Yes	77314	52	0.73	0.67	0.79	<0.001	
Alcohol intake frequency							0.172
None	8732	5.9	1.04	0.78	1.37	0.805	
<3	60714	40.8	0.8	0.72	0.9	<0.001	
>=3	79335	53.3	0.78	0.71	0.85	<0.001	
BMI							0.843

underweight	353	0.2	0.77	0.24	2.45	0.661	
normal	36283	24.4	0.79	0.69	0.89	<0.001	
overweight	73799	49.6	0.77	0.7	0.85	<0.001	
Obesity	38346	25.8	0.72	0.63	0.83	<0.001	
Duration of walks							0.775
<15	12583	8.5	0.71	0.57	0.89	0.002	
<30	36694	24.7	0.72	0.63	0.83	<0.001	
<60	43403	29.2	0.73	0.65	0.83	<0.001	
<180	42821	28.8	0.78	0.69	0.88	<0.001	
>=180	13280	8.9	0.83	0.66	1.04	0.104	
Sleep duration							0.639
<7	36807	24.7	0.73	0.64	0.84	<0.001	
<10	109335	73.5	0.76	0.7	0.82	<0.001	
>=10	2639	1.8	0.62	0.41	0.95	0.029	
Blood pressure							0.142
normal	15739	10.6	0.66	0.54	0.82	<0.001	
High-normal	15646	10.5	0.59	0.47	0.73	<0.001	
Grade 1	38693	26	0.78	0.68	0.89	<0.001	
Grade 2	74225	49.9	0.76	0.69	0.83	<0.001	
Grade 3	4478	3	0.66	0.46	0.93	0.016	
Medication							0.016
Blood pressure medication	15538	10.4	0.71	0.59	0.84	<0.001	
Cholesterol lowering medication	12674	8.5	0.91	0.75	1.11	0.355	
Insulin	266	0.2	2.12	0.59	7.63	0.252	
Mixed	24022	16.1	0.68	0.59	0.78	<0.001	
None of the above	96281	64.7	0.84	0.77	0.92	<0.001	

Code for R

```
library(haven)
library(dplyr)
library(survey)
library(tableone)
library(mice)
library(car)
library(reportReg)
library(writexl)
library(openxlsx)
library(haven)
library(dplyr)
library(tidyr)
library(magrittr)
library(glue)
library(lubridate)
library(survival)
library(magrittr)
library(purrr)
library(jstable)
library(ggrcs)
library(rms)
library(ggplot2)
library(scales)
library(cowplot)
library(survey)
library(bruceR)
library(tidyverse)
library(survRM2)
library(survminer)
#########
getwd()
# setwd("C:\\Users\\wang\\Desktop\\pollution and UKB")
# urology<-read.csv("urinary system.csv")
# BPH<-urology[,c(1,8,9)]
# environment<-read.csv("local environment.csv")
# second<-left_join(BPH,environment,by="Participant.ID")
# save(second,file = "second.rdata")
# characteristic<-read.csv("population characteristic.csv")
# second<-left_join(characteristic,second,by="Participant.ID")
# save(second,file = "second.rdata")
# ######### ####################
getwd()
BPH<-read.csv("BPH.csv")

which(names(BPH)=="Sex")#根据列名查找列的位置

BPH<-BPH[which(BPH$Sex=="Male"),]#

BPH$time<-as.numeric(as.Date(BPH$Date.N40.first.reported..hyperplasia.of.prostate, "%Y-%m-%d")-
as.Date(BPH$Date.of.attending.assessment.centre...Instance.0, "%Y-%m-%d"))
 # BPH<-BPH%>%filter(BPH$time<=0)%>%select()
write.csv(BPH,"BPHfilter.csv")##
BPH1<-read.csv("BPHfilter.csv")
BPH1<-BPH1[,-1]
followtime<-read.csv("population characteristic.csv")
followtime<-followtime[,c(1,32,33,35)]
BPH1<-left_join(BPH1,followtime,by="Participant.ID")
BPH1$time2<-as.numeric(as.Date(BPH1$Date.lost.to.follow.up, "%Y-%m-%d")-
as.Date(BPH1$Date.of.attending.assessment.centre...Instance.0, "%Y-%m-%d"))
write.csv(BPH1,"BPH1.csv")
BPH1<-read.csv("")
BPH1$time3<-as.numeric(as.Date("2023-01-01", "%Y-%m-%d")- as.Date(BPH1$Date.of.attending.assessment.centre...Instance.0,
"%Y-%m-%d"))
BPH1<-dplyr::relocate(BPH1, "time","time2","time3" ,.before = Date.of.last.personal.contact.with.UK.Biobank)
BPH1$time4<-ifelse(!is.na(BPH1$time),BPH1$time,BPH1$time2)
BPH1$finaltime<-ifelse(!is.na(BPH1$time4),BPH1$time4,BPH1$time3)
BPH2<-BPH1
BPH2$BPH<-ifelse(!is.na(BPH2$time),1,0)
BPH2$fustate<-ifelse(!is.na(BPH2$time2),1,0)
write.csv(BPH2,"BPH22.csv")#纳入 215227 中 23278 名患者，BPH 1

BPH3<-read.csv("BPH3(大于 45.csv")
bloodandurine<-read.csv("blood and urine.csv")
BPH3<-left_join(BPH3,bloodandurine,by="Participant.ID")
table(BPH3$BPH)
```

```
can <- colnames(BPH3)[9:77]
BPH3<-BPH3[,-76]
uni_sur <- sapply(can, function(x) as.formula(paste('Surv(time.day., BPH==1)~', x)))
uni_cox <- lapply(uni_sur, function(x){coxph(x, data = BPH3)})
uni_results <- lapply(uni_cox, function(x) {
  # x <- uni_cox$Gender

  x <- summary(x)
  p.value <- signif(x$wald["pvalue"], digits = 2)
  HR <- signif(x$coef[2], digits = 2)
  #
  HR.confint.lower <- signif(x$conf.int[,"lower .95"], digits = 2)
  HR.confint.upper <- signif(x$conf.int[,"upper .95"], digits = 2)
  HR <- paste0(HR, " (", HR.confint.lower, "-", HR.confint.upper, ")")
  res <- c(p.value, HR)
  names(res) <- c("p.value", "HR (95% CI)")
  return(res)
})
res_uni_cox <- as.data.frame(t(as.data.frame(uni_results, check.names = FALSE)))
as.data.frame(res_uni_cox)
write.csv(res_uni_cox,".csv")
pMiss<-function(x){191336-sum(is.na(x))}##
apply(BPH3,2,pMiss)
table()
save(BPH3,file = "BPH3.rdata")
########################## ###########

load("BPH3.rdata")
Covariates<-read.csv("Covariatesnewformice.csv",na.strings="NA") #na.strings=NA，
BPH3<-left_join(BPH3,Covariates,by="Participant.ID")
table(BPH3$Medication.M)
table(is.na(BPH3$Age.when.attended.assessment.centre...Instance.0))
BPH3$age<-BPH3$Age.when.attended.assessment.centre...Instance.0

#FALSE  TRUE
#8854  2602
BPH3$age_class<-dplyr::case_when(BPH3$Age.when.attended.assessment.centre...Instance.0 <50  ~ 1,
                                 BPH3$Age.when.attended.assessment.centre...Instance.0 <60  ~ 2,
                                 BPH3$Age.when.attended.assessment.centre...Instance.0 <70 ~ 3,
                                BPH3$Age.when.attended.assessment.centre...Instance.0 >=70 ~ 4,
                                 is.na(BPH3$Age.when.attended.assessment.centre...Instance.0) ~ NA)
BPH3<-BPH3[,-7]
BPH3<-dplyr::relocate(BPH3, "age","age_class", .before = agefinal)

table(BPH3$age_class)
#1     2     3     4
#29006 70734 90552  1044
colnames(BPH3)
table(is.na(BPH3$Townsend.deprivation.index.at.recruitment))
# fun_quantile <- function(x){
#   dplyr::case_when(
#     x <= quantile(x,na.rm = T)[2] ~ "Q1",   # (,]
#     x <= quantile(x,na.rm = T)[3] ~ "Q2", # (,]
#     x <= quantile(x,na.rm = T)[4] ~ "Q3",   # (,]
#     x <= max(x,na.rm = T) ~ "Q4",          # (,]
#     TRUE ~ NA  # others is missing value
#   )
# }

BPH3$Townsend<-BPH3$Townsend.deprivation.index.at.recruitment
BPH3<-dplyr::relocate(BPH3,"Townsend",.before = Basophill.count...Instance.0)
#Townsend
quantile(BPH3$Townsend,c(0.2,0.4,0.6,0.8),na.rm = TRUE)

BPH3$Townsend<-dplyr::case_when(BPH3$Townsend<=-3.96  ~ 1,
                               BPH3$Townsend<=-2.81  ~ 2,
                               BPH3$Townsend<=-1.34  ~ 3,
                               BPH3$Townsend<=1.35 ~ 4,
                               BPH3$Townsend>1.35 ~ 5,
                               is.na(BPH3$Townsend) ~ NA)
table(is.na(BPH3$Townsend))


BPH3<-dplyr::relocate(BPH3,"Qualifications",.before = Basophill.count...Instance.0)
table(BPH3$Qualifications)
table(is.na(BPH3$Qualifications))#
#NA 4016
#A levels/AS levels or equivalent#19314
#College or University degree #62637
#CSEs or equivalent #9385
#None of the above #34794
#NVQ or HND or HNC or equivalent #17815
#O levels/GCSEs or equivalent #34456
#Other professional qualifications eg: nursing, teaching 8919
```

```
BPH3<-dplyr::relocate(BPH3,"Current.employment.status",.before = Basophill.count...Instance.0)
table(BPH3$Current.employment.status)
table(is.na(BPH3$Current.employment.status))#
#NA 2170
#Doing unpaid or voluntary work                      Full or part-time student
#549                                          279
#In paid employment or self-employed                 Looking after home and/or family
#111635                                            978
#Retired Unable to work because of sickness or disability
#63304                                          8022
#Unemployed
#4399


BPH3<-dplyr::relocate(BPH3,"Ethnic.background",.before = Basophill.count...Instance.0)
table(BPH3$Ethnic.background)
table(is.na(BPH3$Ethnic.background))
#NA 1254
#Asian or Asian British   Black or Black British    Mixed     Other ethnic group
#4420                     2489                  801                  1420
#White
#180952


BPH3<-dplyr::relocate(BPH3,"Smoking.status",.before = Basophill.count...Instance.0)
table(BPH3$Smoking.status)
table(is.na(BPH3$Smoking.status))
#Current                Never Prefer not to answer             Previous
#23310               91240                 846               75572
#NA 368

BPH3<-dplyr::relocate(BPH3,"Alcohol.intake.frequency",.before = Basophill.count...Instance.0)
table(BPH3$Alcohol.intake.frequency)
table(is.na(BPH3$Smoking.status))#
#<3 drinks/week >=3 drinks/week          Never
#78081         100913          11713
#NA 368

#BMI
BPH3<-dplyr::relocate(BPH3,"BMI",.before = Basophill.count...Instance.0)

#BMI
BPH3$BMI<-dplyr::case_when(BPH3$BMI <18.5  ~ 1,#underweight
                           BPH3$BMI <25    ~ 2,#normal
                           BPH3$BMI <30  ~ 3,#overweight
                           BPH3$BMI >=30  ~ 4, #Obesity
                           is.na(BPH3$BMI) ~ NA)

table(is.na(BPH3$BMI))
table(BPH3$BMI)
#FALSE   TRUE
#189955   1381
#1     2     3     4
#464 46541 93800 49150

BPH3<-dplyr::relocate(BPH3,"Duration.of.walks",.before = Basophill.count...Instance.0)

BPH3$Duration.of.walks<-dplyr::case_when(BPH3$Duration.of.walks <15  ~ 1,
                                         BPH3$Duration.of.walks <30  ~ 2,
                                         BPH3$Duration.of.walk <60  ~ 3,
                                         BPH3$Duration.of.walk <180 ~ 4,
                                         BPH3$Duration.of.walks >=180  ~ 5,
                                         is.na(BPH3$Duration.of.walk) ~ NA)

table(BPH3$Duration.of.walks)
#1     2     3     4     5
#14181 40854 48727 47343 14973
table(is.na(BPH3$Duration.of.walks))#
#NA 25258


BPH3<-dplyr::relocate(BPH3,"Sleep.duration",.before = Basophill.count...Instance.0)

BPH3$Sleep.duration<-dplyr::case_when(BPH3$Sleep.duration <7  ~ 1,
                                      BPH3$Sleep.duration <10  ~ 2,
                                      BPH3$Sleep.duration >=10  ~ 3,
                                      is.na(BPH3$Sleep.duration) ~ NA)
table(BPH3$Sleep.duration)
#1      2      3
#47533 138952   3492
table(is.na(BPH3$Sleep.duration))#

#FALSE   TRUE
#189977   1359
```

```
BPH3$Diastolic<-
ifelse(!is.na(BPH3$Diastolic.blood.pressure.automated),BPH3$Diastolic.blood.pressure.automated,BPH3$Diastolic.blood.pressure.
manual)
BPH3$Systolic<-
ifelse(!is.na(BPH3$Systolic.blood.pressure.automated),BPH3$Systolic.blood.pressure.automated,BPH3$Systolic.blood.pressure.m
anual)
BPH3$Systolic<-dplyr::case_when(BPH3$Systolic <120  ~ 1,#normal
                                BPH3$Systolic <130  ~ 2, #High-normal
                                BPH3$Systolic <140  ~ 3, #Grade 1:
                                BPH3$Systolic <180  ~ 4, #Grade 2
                                BPH3$Systolic >=180  ~ 5, #Grade 3
                                is.na(BPH3$Systolic) ~ NA)
BPH3$Diastolic<-dplyr::case_when(BPH3$Diastolic <80  ~ 1,#normal
                            BPH3$Diastolic <90  ~ 3, #Grade 1:
                            BPH3$Diastolic <110  ~ 4, #Grade 2
                            BPH3$Diastolic >=110  ~ 5, #Grade 3
                            is.na(BPH3$Diastolic) ~ NA)

BPH3$bp<-ifelse(BPH3$Systolic>BPH3$Diastolic,BPH3$Systolic,BPH3$Diastolic)
BPH3<-dplyr::relocate(BPH3,"bp",.before = Basophill.count...Instance.0)

table(BPH3$Medication.M)
#Blood pressure medication Cholesterol lowering medication                      Insulin
#19693                            15986                              356
#Mixed              None of the above
#30663                           120906
BPH3<-dplyr::relocate(BPH3,"Medication.M",.before = Basophill.count...Instance.0)


names(BPH3)  #
BPH3<-BPH3[,1:81]

save(BPH3,file = "fourth.rdata")
---------- -----------

getwd()
load("fourth.rdata")

data<-BPH3
data<-data[,c(1:20,55,64:68,74,75,78)]

names(data)
table(is.na(BPH3$Gamma.glutamyltransferase...Instance.0))
data<-
tidyr::drop_na(data,"Gamma.glutamyltransferase...Instance.0","Glucose...Instance.0","Glycated.haemoglobin..HbA1c....Instance.0"
                  ,"HDL.cholesterol...Instance.0","IGF.1...Instance.0"
                  , "SHBG...Instance.0"
                  ,"Testosterone...Instance.0","Triglycerides...Instance.0",   "Apolipoprotein.A...Instance.0" )#

table(data$Duration.of.walks)
data$age_class<-factor(data$age_class,
                    levels = c(1,2,3),
                    labels = c("<50","<60",">=60"))

data$BPH<-factor(data$BPH,
              levels = c(1,0),
              labels = c("Yes","No"))


data$Townsend<-factor(data$Townsend,
                    levels = c(1,2,3,4,5),
                    labels = c("<=-3.96","<=-2.81","<=-1.34","<=1.35",">1.35"))
data$Duration.of.walks<-factor(data$Duration.of.walks,
                      levels = c(1,2,3,4,5),
                      labels = c("<15","<30","<60 ","<180",">=180"))
data$Sleep.duration<-factor(data$Sleep.duration,
               levels = c(1,2,3),
               labels = c("<7","<10",">=10"))

data$BMI<-factor(data$BMI,
                        levels = c(1,2,3,4),
                        labels = c("underweight","normal","overweight","Obesity"))
data$bp<-factor(data$bp,
               levels = c(1,2,3,4,5),
               labels = c("normal","High-normal","Grade 1","Grade 2","Grade 3"))

data$Smoking.status<-factor(data$Smoking.status,
                              levels = c(0,1),
                              labels = c("No","Yes"))

# names(data)
#
# save(data,file = "data.rdata")
#
#
```

```
# load("data.rdata")
# # names(data)
#
# # data<-data[,-c(83,85,87,89,91)]
# # save(data,file = "newdata.rdata")
# myVars <- c( "time.day.", "time.year.","BPH","fustate","Year.of.birth","age","age_class","agefinal","Townsend"
#             , "Qualifications", "Current.employment.status", "Ethnic.background","Smoking.status","Alcohol.intake.frequency","BMI"
#             ,"Duration.of.walks" , "Sleep.duration","bp","Medication.M","Basophill.count...Instance.0",
"Basophill.percentage...Instance.0"
#             ,"Eosinophill.count...Instance.0", "Eosinophill.percentage...Instance.0","Haematocrit.percentage...Instance.0",
"Haemoglobin.concentration...Instance.0"
#             ,"High.light.scatter.reticulocyte.count...Instance.0","High.light.scatter.reticulocyte.percentage...Instance.0","Immature.reticu
locyte.fraction...Instance.0","Lymphocyte.count...Instance.0", "Lymphocyte.percentage...Instance.0"
#             ,"Mean.corpuscular.haemoglobin...Instance.0" ,"Mean.corpuscular.haemoglobin.concentration...Instance.0","Mean.corpus
cular.volume...Instance.0"
#             ,"Mean.platelet..thrombocyte..volume...Instance.0","Mean.reticulocyte.volume...Instance.0","Mean.sphered.cell.volume...I
nstance.0" ,"Monocyte.count...Instance.0","Monocyte.percentage...Instance.0"
#             ,"Neutrophill.count...Instance.0" ,"Neutrophill.percentage...Instance.0","Nucleated.red.blood.cell.count...Instance.0","Nucle
ated.red.blood.cell.percentage...Instance.0"
#             ,"Platelet.count...Instance.0","Platelet.crit...Instance.0",
"Platelet.distribution.width...Instance.0","Red.blood.cell..erythrocyte..count...Instance.0"
#             ,"Red.blood.cell..erythrocyte..distribution.width...Instance.0","Reticulocyte.count...Instance.0","Reticulocyte.percentage...In
stance.0","White.blood.cell..leukocyte..count...Instance.0"
#             ,"Alanine.aminotransferase...Instance.0","Albumin...Instance.0" ,"Alkaline.phosphatase...Instance.0","Apolipoprotein.A...In
stance.0",
"Apolipoprotein.B...Instance.0","Aspartate.aminotransferase...Instance.0","C.reactive.protein...Instance.0","Calcium...Instance.0","C
holesterol...Instance.0","Creatinine...Instance.0"
#             ,"Cystatin.C...Instance.0","Direct.bilirubin...Instance.0","Gamma.glutamyltransferase...Instance.0","Glucose...Instance.0","
Glycated.haemoglobin..HbA1c....Instance.0"
#             ,
"HDL.cholesterol...Instance.0","IGF.1...Instance.0","LDL.direct...Instance.0","Lipoprotein.A...Instance.0","Oestradiol...Instance.0","P
hosphate...Instance.0"
#             ,"Rheumatoid.factor...Instance.0","SHBG...Instance.0","Testosterone...Instance.0","Total.bilirubin...Instance.0","Total.protei
n...Instance.0","Triglycerides...Instance.0"
#             ,"Urate...Instance.0","Urea...Instance.0","Vitamin.D...Instance.0","Total.volume.of.urine.samples.held.by.UKB...Instance.0",
"Creatinine..enzymatic..in.urine...Instance.0"
#             ,"Creatinine..enzymatic..in.urine.result.flag...Instance.0","Microalbumin.in.urine...Instance.0","Microalbumin.in.urine.result.f
lag...Instance.0"
#             ,"Potassium.in.urine...Instance.0","Potassium.in.urine.result.flag...Instance.0","Sodium.in.urine...Instance.0","Sodium.in.uri
ne.result.flag...Instance.0"    )
# # myVars <-
c("age_final","age_class","residence" ,"marital_status","education_level","BMI_a","tobacco_consumption" ,"alcohol_consumption","
physical_activity","Hypertension" ,
# #             "Diabetes","High_cholesterol","Tumor" ,"Chronic_lung_disease" ,"Chronic_heart_diseases" ,
"Stroke" ,"Bone_or_joint_diseases","Neurological_or_psychiatric_problem")
# # catVars<-
c("age_class","depression_class","caste_tribe","residence" ,"marital_status","education_level","BMI_a","tobacco_consumption" ,"al
cohol_consumption","physical_activity","Hypertension" ,
# #            "Diabetes","High_cholesterol","Tumor" ,"Chronic_lung_disease" ,"Chronic_heart_diseases" ,
"Stroke" ,"Bone_or_joint_diseases","Neurological_or_psychiatric_problem")
# catVars<-c("fustate","age_class","Townsend"  ,"Qualifications" ,"Current.employment.status","Ethnic.background",
"Smoking.status",   "Alcohol.intake.frequency",
#            "Duration.of.walks" , "Sleep.duration","Medication.M")
# novars<-c("time.day.", "time.year.","Year.of.birth","age","agefinal"
#          , "BMI","bp","Basophill.count...Instance.0", "Basophill.percentage...Instance.0"
#          ,"Eosinophill.count...Instance.0", "Eosinophill.percentage...Instance.0","Haematocrit.percentage...Instance.0",
"Haemoglobin.concentration...Instance.0"
#          ,"High.light.scatter.reticulocyte.count...Instance.0","High.light.scatter.reticulocyte.percentage...Instance.0","Immature.reticul
ocyte.fraction...Instance.0","Lymphocyte.count...Instance.0", "Lymphocyte.percentage...Instance.0"
#          ,"Mean.corpuscular.haemoglobin...Instance.0" ,"Mean.corpuscular.haemoglobin.concentration...Instance.0","Mean.corpusc
ular.volume...Instance.0"
#          ,"Mean.platelet..thrombocyte..volume...Instance.0","Mean.reticulocyte.volume...Instance.0","Mean.sphered.cell.volume...Ins
tance.0" ,"Monocyte.count...Instance.0","Monocyte.percentage...Instance.0"
#          ,"Neutrophill.count...Instance.0" ,"Neutrophill.percentage...Instance.0","Nucleated.red.blood.cell.count...Instance.0","Nuclea
ted.red.blood.cell.percentage...Instance.0"
#          ,"Platelet.count...Instance.0","Platelet.crit...Instance.0",
"Platelet.distribution.width...Instance.0","Red.blood.cell..erythrocyte..count...Instance.0"
#          ,"Red.blood.cell..erythrocyte..distribution.width...Instance.0","Reticulocyte.count...Instance.0","Reticulocyte.percentage...Ins
tance.0","White.blood.cell..leukocyte..count...Instance.0"
#          ,"Alanine.aminotransferase...Instance.0","Albumin...Instance.0" ,"Alkaline.phosphatase...Instance.0","Apolipoprotein.A...Inst
ance.0",
"Apolipoprotein.B...Instance.0","Aspartate.aminotransferase...Instance.0","C.reactive.protein...Instance.0","Calcium...Instance.0","C
holesterol...Instance.0","Creatinine...Instance.0"
#         ,"Cystatin.C...Instance.0","Direct.bilirubin...Instance.0","Gamma.glutamyltransferase...Instance.0","Glucose...Instance.0","Gl
ycated.haemoglobin..HbA1c....Instance.0"
#         ,
"HDL.cholesterol...Instance.0","IGF.1...Instance.0","LDL.direct...Instance.0","Lipoprotein.A...Instance.0","Oestradiol...Instance.0","P
hosphate...Instance.0"
#          ,"Rheumatoid.factor...Instance.0","SHBG...Instance.0","Testosterone...Instance.0","Total.bilirubin...Instance.0","Total.protein
...Instance.0","Triglycerides...Instance.0"
#          ,"Urate...Instance.0","Urea...Instance.0","Vitamin.D...Instance.0","Total.volume.of.urine.samples.held.by.UKB...Instance.0",
"Creatinine..enzymatic..in.urine...Instance.0"
#          ,"Creatinine..enzymatic..in.urine.result.flag...Instance.0","Microalbumin.in.urine...Instance.0","Microalbumin.in.urine.result.fl
ag...Instance.0"
#          ,"Potassium.in.urine...Instance.0","Potassium.in.urine.result.flag...Instance.0","Sodium.in.urine...Instance.0","Sodium.in.urin
```

```
e.result.flag...Instance.0" )
# #制作 Table 1
# tab1 <- CreateTableOne(data = data,
#                        strata =c("BPH"),    #
#                        vars =myVars,
#                        factorVars =catVars,  #
#                        addOverall=F,
#                        )
# Tablel <- print(tab1, showAllLevels = TRUE,#
#                 cramVars = catVars,       #
#                 nonnormal = novars,#
#                 #exact ="M", #
#                 catDigits=2,#
#                 contDigits=2,#
#                 quote = FALSE,#
#                 noSpace=TRUE,#
#
#                 printToggle = FALSE#输出 matrix
# )
# write.csv(Tablel,"Table.csv",quote=TRUE,row.names=TRUE
# cols <- colnames(data[,c(2:3,7,9,16,19:20,22:82,84,86,88,90)])
#
# multi_shapiro <- function(cols, group, data) {
#   result <- data.frame('Characteristics'='','P1'=0,'P2'=0, 'NormalDistribution' = '')
#   for (col in cols) {
#     shapiro <- tapply(data[[col]],data[[group]],ks.test)
#     isNormal = ifelse(shapiro[[1]]$p.value > 0.05 & shapiro[[2]]$p.value > 0.05, 'true', 'false')
#     row = c(col, shapiro[[1]]$p.value, shapiro[[2]]$p.value, isNormal)
#     result = rbind(result, row)
#   }
#   result = result[-1,]
#   return(result)
# }
# pick_nd_vars <- function(res) {
#   return(res$Characteristics[res$NormalDistribution == 'true'])
# }
#
#
# res <- multi_shapiro(cols,"Type",dataa)
colnames<-c(2:3,7,9,16,19:20,22:82,84,86,88,90)
results <- lapply(data[colnames], function(x) ks.test(x, "pnorm"))
class(results)
ks.test(data$Triglycerides...Instance.0,"pnorm")
leveneTest(data$depression_quality,data$BPH)# p<0.05
data$Triglycerides...Instance.0
#K-W
kruskal.test(Triglycerides...Instance.0~BPH,data=data)
pMiss<-function(x){sum(is.na(x))}##找 NA
apply(data_mice,2,pMiss)
load("fourth.rdata")
data_mice<-BPH3
data_mice<-data_mice[,c(1:20,55,64:68,74,75,78)]####

names(data_mice)
data_mice<-
tidyr::drop_na(data_mice,"Gamma.glutamyltransferase...Instance.0","Glucose...Instance.0","Glycated.haemoglobin..HbA1c....Instan
ce.0"
                   ,"HDL.cholesterol...Instance.0","IGF.1...Instance.0"
                   , "SHBG...Instance.0"
                   ,"Testosterone...Instance.0","Triglycerides...Instance.0",   "Apolipoprotein.A...Instance.0"
                 )#

data_mice1 <- mice(data_mice,meth="rf",seed=500)
data_mice#
# data_mice$imp #
# data_mice$imp$BMI
# impt<-mice(diabetes,maxit=30, seed=20221113,print = FALSE)
# plot(impt)
data_mice<- complete(data_mice1)##rf，qQualifications  Current.employment.status  Ethnic.background  Medication.M
data_mice2<-tidyr::drop_na(data_mice,"Qualifications","Current.employment.status","Ethnic.background","Medication.M")#148781
save(data_mice2,file = "data_mice2.rdata")
load("data_mice2.rdata")

data_mice2<-data_mice2[,1:2]

data<-left_join(data_mice2,data,by="Participant.ID")
data<-data[,-2]

myVars <- c( "time.day..y", "time.year.","BPH","fustate","Year.of.birth","age","age_class","agefinal","Townsend"
            , "Qualifications", "Current.employment.status", "Ethnic.background","Smoking.status","Alcohol.intake.frequency","BMI"
            ,"Duration.of.walks" , "Sleep.duration","bp","Medication.M","Basophill.count...Instance.0",
"Basophill.percentage...Instance.0"
            ,"Eosinophill.count...Instance.0", "Eosinophill.percentage...Instance.0","Haematocrit.percentage...Instance.0",
"Haemoglobin.concentration...Instance.0"
            ,"High.light.scatter.reticulocyte.count...Instance.0","High.light.scatter.reticulocyte.percentage...Instance.0","Immature.reticul
```

```
ocyte.fraction...Instance.0","Lymphocyte.count...Instance.0", "Lymphocyte.percentage...Instance.0"
            ,"Mean.corpuscular.haemoglobin...Instance.0" ,"Mean.corpuscular.haemoglobin.concentration...Instance.0","Mean.corpusc
ular.volume...Instance.0"
            ,"Mean.platelet..thrombocyte..volume...Instance.0","Mean.reticulocyte.volume...Instance.0","Mean.sphered.cell.volume...Ins
tance.0" ,"Monocyte.count...Instance.0","Monocyte.percentage...Instance.0"
            ,"Neutrophill.count...Instance.0" ,"Neutrophill.percentage...Instance.0","Nucleated.red.blood.cell.count...Instance.0","Nuclea
ted.red.blood.cell.percentage...Instance.0"
            ,"Platelet.count...Instance.0","Platelet.crit...Instance.0",
"Platelet.distribution.width...Instance.0","Red.blood.cell..erythrocyte..count...Instance.0"
            ,"Red.blood.cell..erythrocyte..distribution.width...Instance.0","Reticulocyte.count...Instance.0","Reticulocyte.percentage...Ins
tance.0","White.blood.cell..leukocyte..count...Instance.0"
            ,"Alanine.aminotransferase...Instance.0","Albumin...Instance.0" ,"Alkaline.phosphatase...Instance.0","Apolipoprotein.A...Inst
ance.0",
"Apolipoprotein.B...Instance.0","Aspartate.aminotransferase...Instance.0","C.reactive.protein...Instance.0","Calcium...Instance.0","C
holesterol...Instance.0","Creatinine...Instance.0"
            ,"Cystatin.C...Instance.0","Direct.bilirubin...Instance.0","Gamma.glutamyltransferase...Instance.0","Glucose...Instance.0","G
lycated.haemoglobin..HbA1c....Instance.0"
            ,
"HDL.cholesterol...Instance.0","IGF.1...Instance.0","LDL.direct...Instance.0","Lipoprotein.A...Instance.0","Oestradiol...Instance.0","P
hosphate...Instance.0"
            ,"Rheumatoid.factor...Instance.0","SHBG...Instance.0","Testosterone...Instance.0","Total.bilirubin...Instance.0","Total.protein
...Instance.0","Triglycerides...Instance.0"
            ,"Urate...Instance.0","Urea...Instance.0","Vitamin.D...Instance.0","Total.volume.of.urine.samples.held.by.UKB...Instance.0",
"Creatinine..enzymatic..in.urine...Instance.0"
            ,"Creatinine..enzymatic..in.urine.result.flag...Instance.0","Microalbumin.in.urine...Instance.0","Microalbumin.in.urine.result.fl
ag...Instance.0"
            ,"Potassium.in.urine...Instance.0","Potassium.in.urine.result.flag...Instance.0","Sodium.in.urine...Instance.0","Sodium.in.urin
e.result.flag...Instance.0"    )
# myVars <-
c("age_final","age_class","residence" ,"marital_status","education_level","BMI_a","tobacco_consumption" ,"alcohol_consumption","
physical_activity","Hypertension" ,
#             "Diabetes","High_cholesterol","Tumor" ,"Chronic_lung_disease" ,"Chronic_heart_diseases" ,
"Stroke" ,"Bone_or_joint_diseases","Neurological_or_psychiatric_problem")
#
# catVars<-
c("age_class","depression_class","caste_tribe","residence" ,"marital_status","education_level","BMI_a","tobacco_consumption" ,"al
cohol_consumption","physical_activity","Hypertension" ,
#            "Diabetes","High_cholesterol","Tumor" ,"Chronic_lung_disease" ,"Chronic_heart_diseases" ,
"Stroke" ,"Bone_or_joint_diseases","Neurological_or_psychiatric_problem")
catVars<-c("fustate","age_class","Townsend"  ,"Qualifications" ,"Current.employment.status","Ethnic.background",
"Smoking.status",   "Alcohol.intake.frequency",
           "Duration.of.walks" , "Sleep.duration","Medication.M")
novars<-c("time.day..y", "time.year.","Year.of.birth","age","agefinal"
         , "BMI","bp","Basophill.count...Instance.0", "Basophill.percentage...Instance.0"
         ,"Eosinophill.count...Instance.0", "Eosinophill.percentage...Instance.0","Haematocrit.percentage...Instance.0",
"Haemoglobin.concentration...Instance.0"
         ,"High.light.scatter.reticulocyte.count...Instance.0","High.light.scatter.reticulocyte.percentage...Instance.0","Immature.reticuloc
yte.fraction...Instance.0","Lymphocyte.count...Instance.0", "Lymphocyte.percentage...Instance.0"
         ,"Mean.corpuscular.haemoglobin...Instance.0" ,"Mean.corpuscular.haemoglobin.concentration...Instance.0","Mean.corpuscul
ar.volume...Instance.0"
         ,"Mean.platelet..thrombocyte..volume...Instance.0","Mean.reticulocyte.volume...Instance.0","Mean.sphered.cell.volume...Insta
nce.0" ,"Monocyte.count...Instance.0","Monocyte.percentage...Instance.0"
         ,"Neutrophill.count...Instance.0" ,"Neutrophill.percentage...Instance.0","Nucleated.red.blood.cell.count...Instance.0","Nucleate
d.red.blood.cell.percentage...Instance.0"
         ,"Platelet.count...Instance.0","Platelet.crit...Instance.0",
"Platelet.distribution.width...Instance.0","Red.blood.cell..erythrocyte..count...Instance.0"
         ,"Red.blood.cell..erythrocyte..distribution.width...Instance.0","Reticulocyte.count...Instance.0","Reticulocyte.percentage...Insta
nce.0","White.blood.cell..leukocyte..count...Instance.0"
         ,"Alanine.aminotransferase...Instance.0","Albumin...Instance.0" ,"Alkaline.phosphatase...Instance.0","Apolipoprotein.A...Insta
nce.0",
"Apolipoprotein.B...Instance.0","Aspartate.aminotransferase...Instance.0","C.reactive.protein...Instance.0","Calcium...Instance.0","C
holesterol...Instance.0","Creatinine...Instance.0"
         ,"Cystatin.C...Instance.0","Direct.bilirubin...Instance.0","Gamma.glutamyltransferase...Instance.0","Glucose...Instance.0","Gly
cated.haemoglobin..HbA1c....Instance.0"
         ,
"HDL.cholesterol...Instance.0","IGF.1...Instance.0","LDL.direct...Instance.0","Lipoprotein.A...Instance.0","Oestradiol...Instance.0","P
hosphate...Instance.0"
         ,"Rheumatoid.factor...Instance.0","SHBG...Instance.0","Testosterone...Instance.0","Total.bilirubin...Instance.0","Total.protein...
Instance.0","Triglycerides...Instance.0"
         ,"Urate...Instance.0","Urea...Instance.0","Vitamin.D...Instance.0","Total.volume.of.urine.samples.held.by.UKB...Instance.0",
"Creatinine..enzymatic..in.urine...Instance.0"
         ,"Creatinine..enzymatic..in.urine.result.flag...Instance.0","Microalbumin.in.urine...Instance.0","Microalbumin.in.urine.result.flag
...Instance.0"
         ,"Potassium.in.urine...Instance.0","Potassium.in.urine.result.flag...Instance.0","Sodium.in.urine...Instance.0","Sodium.in.urine.
result.flag...Instance.0" )
#Table 1
tab1 <- CreateTableOne(data = data,
                      strata =c("BPH"),    #
                      vars =myVars,       #
                      factorVars =catVars,
                      addOverall=F,
)      #
?CreateTableOne
TableI <- print(tab1, showAllLevels = TRUE,
               cramVars = catVars,       #
```

```
                nonnormal = novars,#设
                #exact ="M", #
                catDigits=2,#
                contDigits=2,
                quote = FALSE,#
                noSpace=TRUE,
                #
                printToggle = FALSE,
                showAllLevels = TRUE
)
write.csv(Tablel,"Table.csv",quote=TRUE,row.names=TRUE)

#######

load("data_mice2.rdata")
data_mice2$age_class<-factor(data_mice2$age_class,
                       levels = c(1,2,3,4),
                       labels = c("<50","<60","<70",">=70"))

data_mice2$BPH<-factor(data_mice2$BPH,
                 levels = c(1,0),
                 labels = c("Yes","No"))


data_mice2$Townsend<-factor(data_mice2$Townsend,
                      levels = c(1,2,3,4,5),
                      labels = c("<=-3.96","<=-2.81","<=-1.34","<=1.35",">1.35"))
data_mice2$Duration.of.walks<-factor(data_mice2$Duration.of.walks,
                               levels = c(1,2,3,4,5),
                               labels = c("<15","<30","<60 ","<180",">=180"))
data_mice2$Sleep.duration<-factor(data_mice2$Sleep.duration,
                            levels = c(1,2,3),
                            labels = c("<7","<10",">=10"))

data_mice2$BMI<-factor(data_mice2$BMI,
                 levels = c(1,2,3,4),
                 labels = c("underweight","normal","overweight","Obesity"))
data_mice2$bp<-factor(data_mice2$bp,
                levels = c(1,2,3,4,5),
                labels = c("normal","High-normal","Grade 1","Grade 2","Grade 3"))

data_mice2$Smoking.status<-factor(data_mice2$Smoking.status,
                                  levels = c(0,1),
                                  labels = c("No","Yes"))
myVars <- c( "time.day.", "time.year.","BPH","fustate","Year.of.birth","age","age_class","agefinal","Townsend"
             , "Qualifications", "Current.employment.status", "Ethnic.background","Smoking.status","Alcohol.intake.frequency","BMI"
             ,"Duration.of.walks" , "Sleep.duration","bp","Medication.M","Basophill.count...Instance.0",
"Basophill.percentage...Instance.0"
             ,"Eosinophill.count...Instance.0", "Eosinophill.percentage...Instance.0","Haematocrit.percentage...Instance.0",
"Haemoglobin.concentration...Instance.0"
             ,"High.light.scatter.reticulocyte.count...Instance.0","High.light.scatter.reticulocyte.percentage...Instance.0","Immature.reticul
ocyte.fraction...Instance.0","Lymphocyte.count...Instance.0", "Lymphocyte.percentage...Instance.0"
             ,"Mean.corpuscular.haemoglobin...Instance.0" ,"Mean.corpuscular.haemoglobin.concentration...Instance.0","Mean.corpusc
ular.volume...Instance.0"
             ,"Mean.platelet..thrombocyte..volume...Instance.0","Mean.reticulocyte.volume...Instance.0","Mean.sphered.cell.volume...Ins
tance.0" ,"Monocyte.count...Instance.0","Monocyte.percentage...Instance.0"
             ,"Neutrophill.count...Instance.0" ,"Neutrophill.percentage...Instance.0","Nucleated.red.blood.cell.count...Instance.0","Nuclea
ted.red.blood.cell.percentage...Instance.0"
             ,"Platelet.count...Instance.0","Platelet.crit...Instance.0",
"Platelet.distribution.width...Instance.0","Red.blood.cell..erythrocyte..count...Instance.0"
             ,"Red.blood.cell..erythrocyte..distribution.width...Instance.0","Reticulocyte.count...Instance.0","Reticulocyte.percentage...Ins
tance.0","White.blood.cell..leukocyte..count...Instance.0"
             ,"Alanine.aminotransferase...Instance.0","Albumin...Instance.0" ,"Alkaline.phosphatase...Instance.0","Apolipoprotein.A...Inst
ance.0",
"Apolipoprotein.B...Instance.0","Aspartate.aminotransferase...Instance.0","C.reactive.protein...Instance.0","Calcium...Instance.0","C
holesterol...Instance.0","Creatinine...Instance.0"
             ,"Cystatin.C...Instance.0","Direct.bilirubin...Instance.0","Gamma.glutamyltransferase...Instance.0","Glucose...Instance.0","G
lycated.haemoglobin..HbA1c....Instance.0"
             ,
"HDL.cholesterol...Instance.0","IGF.1...Instance.0","LDL.direct...Instance.0","Lipoprotein.A...Instance.0","Oestradiol...Instance.0","P
hosphate...Instance.0"
             ,"Rheumatoid.factor...Instance.0","SHBG...Instance.0","Testosterone...Instance.0","Total.bilirubin...Instance.0","Total.protein
...Instance.0","Triglycerides...Instance.0"
             ,"Urate...Instance.0","Urea...Instance.0","Vitamin.D...Instance.0","Total.volume.of.urine.samples.held.by.UKB...Instance.0",
"Creatinine..enzymatic..in.urine...Instance.0"
             ,"Creatinine..enzymatic..in.urine.result.flag...Instance.0","Microalbumin.in.urine...Instance.0","Microalbumin.in.urine.result.fl
ag...Instance.0"
             ,"Potassium.in.urine...Instance.0","Potassium.in.urine.result.flag...Instance.0","Sodium.in.urine...Instance.0","Sodium.in.urin
e.result.flag...Instance.0"    )
#
----------### #########
load("data_mice2.rdata")
data_mice2$age_class<-factor(data_mice2$age_class,
                             levels = c(1,2,3,4),
                             labels = c("<50","<60",">=60",">=60"))
```

```
data_mice2$BPH<-factor(data_mice2$BPH,
                       levels = c(1,0),
                       labels = c("Yes","No"))
data_mice2$Townsend<-factor(data_mice2$Townsend,
                            levels = c(1,2,3,4,5),
                            labels = c("<=-3.96","<=-2.81","<=-1.34","<=1.35",">1.35"))
data_mice2$Duration.of.walks<-factor(data_mice2$Duration.of.walks,
                                     levels = c(1,2,3,4,5),
                                     labels = c("<15","<30","<60 ","<180",">=180"))
data_mice2$Sleep.duration<-factor(data_mice2$Sleep.duration,
                                  levels = c(1,2,3),
                                  labels = c("<7","<10",">=10"))
data_mice2$BMI<-factor(data_mice2$BMI,
                       levels = c(1,2,3,4),
                       labels = c("underweight","normal","overweight","Obesity"))
data_mice2$bp<-factor(data_mice2$bp,
                      levels = c(1,2,3,4,5),
                      labels = c("normal","High-normal","Grade 1","Grade 2","Grade 3"))
data_mice2$Smoking.status<-factor(data_mice2$Smoking.status,
                      levels = c(0,1),
                      labels = c("No","Yes"))
data_mice2$Alcohol.intake.frequency<-factor(data_mice2$Alcohol.intake.frequency,
                                  levels = c(0,1,2),
                                  labels = c("None","<3",">=3"))
data_mice<-data_mice2
save(data_mice,file = ".rdata")
load(".rdata")
# names(data_mice)
# data_mice %>%
#   select(Basophill.count...Instance.0) %>%
#   mutate(Basophill.count_z=scale(Basophill.count...Instance.0)) %>%
#   summary()
# data_mice<-mutate(data_mice,Albumin=scale(data_mice$Albumin...Instance.0))
# summary(data_mice$Albumin)
mean(data_mice$time.year.)
can <- colnames(data_mice)[21:29]
uni_sur <- sapply(can, function(x) as.formula(paste('Surv(time.day., BPH=="Yes")~', x)))
uni_cox <- lapply(uni_sur, function(x){coxph(x, data = data_mice)})
uni_results <- lapply(uni_cox, function(x) {
  # x <- uni_cox$Gender
  x <- summary(x)
  p.value <- signif(x$wald["pvalue"], digits = 2)
  HR <- signif(x$coef[2], digits = 2)
  HR.confint.lower <- signif(x$conf.int[,"lower .95"], digits = 2)
  HR.confint.upper <- signif(x$conf.int[,"upper .95"], digits = 2)
  HR <- paste0(HR, " (", HR.confint.lower, "-", HR.confint.upper, ")")

  res <- c(p.value, HR)
  names(res) <- c("p.value", "HR (95% CI)")
  return(res)
})
res_uni_cox <- as.data.frame(t(as.data.frame(uni_results, check.names = FALSE)))
as.data.frame(res_uni_cox)
write.csv(res_uni_cox,".csv")
RMS<- rmst2(time.day..y, BPH,arm,tau=365*3);RMS
?rmst2
data_mice<-data
names(data_mice)
res.cox <- coxph(Surv(time.day..y, BPH=="1")~data_mice$Cystatin.C...Instance.0
             , data = data_mice,id=Participant.ID)
summary(res.cox)
cox.zph(res.cox)
Direct.bilirubin...Instance.0
# age  "Townsend" "Qualifications""Current.employment.status" "Ethnic.background"
names(data_mice)
res.cox <- coxph(Surv(time.day..y, BPH=="1")~data_mice$Urea...Instance.0
+strata(age_class)
+Townsend+Qualifications+Ethnic.background, data = data_mice,id=Participant.ID)
summary(res.cox)
cox.zph(res.cox)
names(data_mice)
res.cox <- coxph(Surv(time.day..y, BPH=="1")~data_mice$HDL.cholesterol...Instance.0
+strata(age_class)
+Townsend+Qualifications+Ethnic.background+Smoking.status+Alcohol.intake.frequency
+BMI+Duration.of.walks+Sleep.duration+bp+Medication.M, data = data_mice,id=Participant.ID)
summary(res.cox)
cox.zph(res.cox)
#P for trend############
names(data_mice)
num=4  ##
xname="age"  ##
xname.group <- paste(xname,'.group',sep='')##
xname.group.2 <- paste(xname,'..group.median',sep='')##
quantile(data_mice[,xname])
```

```
data_mice[,xname.group]<-cut(data_mice[,xname],breaks=c(-Inf,24.0 ,33.5,50.8 ,Inf),labels = c("0","1","2","3"))
breaks<-quantile(data_mice[,xname])
breaks
newcode <- tapply(data_mice[,xname],data_mice[,xname.group],function(z) median(z,na.rm=TRUE))
data_mice[,xname.group.2] <- newcode[1]
for (i in 2:length(newcode)) {data_mice[,xname.group.2][data_mice[,xname.group]==names(newcode)[i]] <- newcode[i]}
label <- rep(NA,num)
if(num>2){
  for (i in 2:length(breaks)) {
    label[i-1] <- paste('≥',breaks[i-1],'&','<',breaks[i],sep='')
    if(i==length(breaks)){
      label[i-1] <- paste('≥',breaks[i-1],'&','≤',breaks[i]-0.01,sep='')
    }
  }
}
counts_summary <- summary(data_mice[,xname.group])
print(counts_summary)
summary(data_mice[,xname.group])
total <- length(data_mice[,xname.group])
percentages_summary <- (counts_summary / total) * 100
print(percentages_summary)
table(data_mice[,xname.group.2])
glm.m <- coxph(Surv(time.day., BPH=="Yes") ~Gamma.glutamyltransferase...Instance.0.group, data = data_mice,id=Participant.ID)
summary(glm.m)
#p for trend
glm.m1 <- coxph(Surv(time.day., BPH=="Yes") ~ Gamma.glutamyltransferase...Instance.0..group.median, data =
data_mice,id=Participant.ID)
summary(glm.m1)
   age  "Townsend" "Qualifications" "Ethnic.background"
names(data_mice)
glm.m <- coxph(Surv(time.day., BPH=="Yes") ~Gamma.glutamyltransferase...Instance.0.group+strata(age_class)
               +Townsend+Qualifications+Ethnic.background, data = data_mice,id=Participant.ID)
summary(glm.m)
glm.m1 <- coxph(Surv(time.day., BPH=="Yes") ~Gamma.glutamyltransferase...Instance.0..group.median++strata(age_class)
                +Townsend+Qualifications+Ethnic.background, data = data_mice,id=Participant.ID)
summary(glm.m1)
names(data_mice)
glm.m <- coxph(Surv(time.day., BPH=="Yes") ~Gamma.glutamyltransferase...Instance.0.group+strata(age_class)

+Townsend+Qualifications+Ethnic.background+Smoking.status+Alcohol.intake.frequency+BMI+Duration.of.walks+Sleep.duration+
bp+Medication.M, data = data_mice,id=Participant.ID)
summary(glm.m)
glm.m1 <- coxph(Surv(time.day., BPH=="Yes") ~Gamma.glutamyltransferase...Instance.0..group.median+strata(age_class)

+Townsend+Qualifications+Ethnic.background+Smoking.status+Alcohol.intake.frequency+BMI+Duration.of.walks+Sleep.duration+
bp+Medication.M, data = data_mice,id=Participant.ID)
summary(glm.m1)

data_mice<-data_mice[,-c(9,12)]

names(data_mice)
res <- TableSubgroupMultiCox(
  formula = Surv(time.day., BPH=="Yes") ~ IGF.1...Instance.0,
  var_subgroups =
c("age_class","Townsend","Qualifications","Ethnic.background","Smoking.status","Alcohol.intake.frequency","BMI","Duration.of.walk
s"
                  ,"Sleep.duration","bp","Medication.M"),
  data = data_mice
)
res
write.csv(res,"IGF.1.csv")
res.cox <- coxph(Surv(time.day., BPH=="Yes")~HDL.cholesterol...Instance.0
                 +strata(age_class)

+Townsend+Qualifications+Ethnic.background+Smoking.status+Alcohol.intake.frequency+BMI+Duration.of.walks+Sleep.duration+
bp+Medication.M, data = data_mice,id=Participant.ID)
# names(data_mice)
# table(data_mice$Medication.M)
# res.cox <- coxph(Surv(time.day., BPH=="Yes")~HDL.cholesterol...Instance.0
+Townsend+Qualifications+Ethnic.background+Smoking.status+Alcohol.intake.frequency+BMI+Duration.of.walks+Sleep.duration+
bp+Medication.M,
#                  data = data_mice[data_mice$age_class == "<60",],id=Participant.ID)
# broom::tidy(res.cox,exponentiate = T,conf.int = T)
# names(df)
# df1 <- df %>%
#   pivot_longer(cols = 8:18,names_to = "var",values_to = "value") %>%
#   arrange(var)
# head(df1)
# ress <- df1 %>%
#   #group_by(var,value) %>%
#   group_nest(var,value) %>%
#   drop_na(value) %>%
#   mutate(#sample_size=map(data, ~ nrow(.x)),
#     model=map(data, ~ coxph(Surv(time.day., BPH=="Yes") ~ HDL.cholesterol...Instance.0,data = df)),
#     res = map(model, broom::tidy,conf.int = T, exponentiate = T)
```

```
#   ) %>%
#   dplyr::select(var,value,res)
# glimpse(ress)
# ss <- df1 %>%
#   group_by(var,value,HDL.cholesterol...Instance.0) %>%
#   drop_na(value) %>%
#   summarise(sample_size=n()) %>%
#   dplyr::select(var,value,HDL.cholesterol...Instance.0,sample_size)
#
# resss <- ress %>%
#   left_join(ss,b=c("var","value")) %>%
#   unnest(res,HDL.cholesterol...Instance.0,sample_size) %>%
#   pivot_wider(names_from = "HDL.cholesterol...Instance.0",values_from = "sample_size",names_prefix =
"HDL.cholesterol...Instance.0_") %>%
#   select(-c(term,std.error,statistic)) %>%
#   mutate(across(where(is.numeric), round,digits=2)) %>%
#   mutate(`HR(95%CI)`=paste0(estimate,"(",conf.low,"-",conf.high,")"))
# head(resss)
# fit <- coxph(Surv(time.day., BPH=="Yes") ~ HDL.cholesterol...Instance.0, data = df)
# res_all <- broom::tidy(fit,exponentiate = T,conf.int = T)
# df %>% count(HDL.cholesterol...Instance.0)
# res_all <- res_all %>%
#   mutate(var="All people",
#          value=" ",
#          rx_0=304,
#          rx_1=305,
#          across(where(is.numeric), round,digits=2)
#   ) %>%
#   mutate(`HR(95%CI)`=paste0(estimate,"(",conf.low,"-",conf.high,")")
#   ) %>%
#   select(var,value,estimate,p.value,conf.low,conf.high,rx_0,rx_1,`HR(95%CI)`)
# res_all
# resss <- bind_rows(res_all,resss)
# head(resss)
# write.csv(resss, file = "resss.csv",quote = F,row.names = T)
library(ggplot2)
#install.packages('rms')
library(rms)
library(ggrcs)
dd<-datadist(data_mice)##rs
options(datadist='dd')
names(data_mice)
 fit <- cph(Surv(time.day., BPH=="Yes")~rcs(HDL.cholesterol...Instance.0,5)
                 +strat(age_class)
+Townsend+Qualifications+Ethnic.background+Smoking.status+Alcohol.intake.frequency+BMI+Duration.of.walks+Sleep.duration+
bp+Medication.M,data=data_mice)
# ggrcs(data=dt,fit=fit,x="HDL.cholesterol...Instance.0")#
anova(fit)
HR<-Predict(fit,HDL.cholesterol...Instance.0,fun=exp,ref.zero = TRUE)
head(HR)
names(data_mice)
ggplot()+
   geom_line(data=HR, aes(HDL.cholesterol...Instance.0,yhat),
            linetype="solid",size=1.5,alpha = 0.7,colour="#0070b9")+
   geom_ribbon(data=HR,
              aes(HDL.cholesterol...Instance.0,ymin = lower, ymax = upper),
              alpha = 0.1,fill="#0070b9")+
   theme_classic()+
   geom_hline(yintercept=1, linetype=2,size=1.2)+
   geom_vline(xintercept=1.2317535,size=1.5,color = '#d40e8c')+
     labs(title = "BPH Risk", x="γ-glutamyltransferase", y="HR (95%CI)")
```
